# Microbial signatures of protected and impacted Northern Caribbean reefs: changes from Cuba to the Florida Keys

**DOI:** 10.1111/1462-2920.14870

**Published:** 2019-12-11

**Authors:** Laura Weber, Patricia González‐Díaz, Maickel Armenteros, Víctor M. Ferrer, Fernando Bretos, Erich Bartels, Alyson E. Santoro, Amy Apprill

**Affiliations:** ^1^ Marine Chemistry and Geochemistry Department Woods Hole Oceanographic Institution Woods Hole MA USA; ^2^ MIT‐WHOI Joint PhD Program in Biological Oceanography Cambridge MA USA; ^3^ Centro de Investigaciones Marinas Universidad de La Habana Habana Cuba; ^4^ Instituto de Ciencias del Mar y Limnología Universidad Nacional Autónoma de México Ciudad México Mexico; ^5^ Phillip and Patricia Frost Museum of Science Miami FL USA; ^6^ Mote Marine Laboratory Florida Keys FL USA; ^7^ Department of Ecology, Evolution and Marine Biology University of California Santa Barbara CA USA

## Abstract

There are a few baseline reef‐systems available for understanding the microbiology of healthy coral reefs and their surrounding seawater. Here, we examined the seawater microbial ecology of 25 Northern Caribbean reefs varying in human impact and protection in Cuba and the Florida Keys, USA, by measuring nutrient concentrations, microbial abundances, and respiration rates as well as sequencing bacterial and archaeal amplicons and community functional genes. Overall, seawater microbial composition and biogeochemistry were influenced by reef location and hydrogeography. Seawater from the highly protected ‘crown jewel’ offshore reefs in Jardines de la Reina, Cuba had low concentrations of nutrients and organic carbon, abundant *Prochlorococcus*, and high microbial community alpha diversity. Seawater from the less protected system of Los Canarreos, Cuba had elevated microbial community beta‐diversity whereas waters from the most impacted nearshore reefs in the Florida Keys contained high organic carbon and nitrogen concentrations and potential microbial functions characteristic of microbialized reefs. Each reef system had distinct microbial signatures and within this context, we propose that the protection and offshore nature of Jardines de la Reina may preserve the oligotrophic paradigm and the metabolic dependence of the community on primary production by picocyanobacteria.

## Introduction

Caribbean coral reefs have undergone dramatic changes over the past 35 years. The collective impacts of climate change, overfishing, and coastal development have caused shifts in functioning and energy transfer in coral reef ecosystems and these changes have been documented at the level of macro‐organisms (Carpenter, 1988; Gardner *et al*., 2003; Miller *et al*., 2009; Valdivia *et al*., 2017). In contrast, the impacts of these stressors on reef microbial communities have not been comprehensively documented because molecular techniques for characterizing uncultivated microbes were unavailable prior to the widespread decline of Caribbean coral reefs. This has led to critical gaps in our understanding of how microorganisms, the smallest and most abundant members of Caribbean coral reefs, have changed in abundance, composition, and function alongside broader ecosystem changes.

Global studies have shown that reefs harbour distinct microbial taxa and genomic adaptations compared to cells found in off‐reef waters (Nelson *et al*., 2011; Kelly *et al*., 2014), suggesting that unique microbial processes occur on coral reefs. Additionally, human impacts (overfishing, pollution) may lead to shifts in reef trophic structure that favour microbial growth (Jackson *et al*., 2001). On coral reefs, this process of ‘microbialization’ begins when grazing pressure on algae is lessened due to the removal of herbivorous fish and sea urchins (Hughes *et al*., 2007; McDole *et al*., 2012; Haas *et al*., 2016). Removal of grazers leads to increases in macroalgae (Hughes *et al*., 2007). More macroalgae may then lead to increases in the standing stock of dissolved organic carbon (DOC) within the water column, increases in the abundance, respiration, and virulence/pathogenicity of heterotrophic microbes, and a net drawdown of DOC (Haas *et al*., 2016). This mechanistic model is referred to as the DDAM (DOC, disease, algae, and microbes) model and has been suggested as one of the invisible causes for the global degradation of coral reefs (Barott and Rohwer, 2012; Haas *et al*., 2016). Despite the attention dedicated to understanding the microbiology of declining coral reef ecosystems, there are still numerous unknowns surrounding the microbiology supporting healthier coral reefs, especially within the Caribbean, which harbour distinct and less diverse coral communities compared to Indo‐Pacific reefs.

Jardines de la Reina (JR) is a protected reef‐system in Cuba that may provide useful insights into the microbial ecology of relatively healthy Caribbean coral reefs. The reefs of JR were historically protected from human activities due to their remote nature and are now further protected because maritime traffic, fishing, and recreational diving and tourism are limited within the boundary of a Marine National Park (est. in 1996) that encapsulates most of the archipelago. Additionally, Cuba does not currently have large‐scale industrialized agriculture or extensive development along most of its coastline (Galford *et al*., 2018; González‐Díaz *et al*., 2018), minimizing the degree to which nutrient run‐off and sedimentation may impact the surrounding waters. The Ana Maria Gulf, referred to here as the JR gulf, spans the inner sea between the island of Cuba and JR. This gulf is populated by small mangrove keys, extensive seagrass meadows, and unvegetated sea beds. These features within the JR gulf have likely reduced pollution as well as human‐induced sedimentation and eutrophication (Galford *et al*., 2018; González‐Díaz *et al*., 2018). Together, the protection of JR from human impacts as well as the ecological services provided by mangrove and seagrass biomes (Mumby *et al*., 2004; Guannel *et al*., 2016) have likely buffered JR from direct human‐induced stressors that plague other reefs in the Caribbean. As a result, this reef‐system is regarded as a ‘crown jewel’ because it supports some of the highest fish biomass (including top predators like sharks and groupers) and coral cover in the Caribbean (Valdivia *et al*., 2017; González‐Díaz *et al*., 2018).

The more impacted reef‐system of Los Canarreos (CAN), Cuba lies ~230 km to the west of JR. CAN encompasses three important keys that have less stringent protection compared to JR: Cayo Largo is an Ecological Reserve and the Rosario and Cantiles Keys are Faunal Refuges. Due to increased accessibility, reefs within CAN are more impacted by humans through subsistence and illegal fishing, tourism, and the diving industry compared to the remote and protected reefs within JR. Fishing has resulted in overexploitation of important finfish and invertebrates in most of Cuba's waters with the exception of the central area within JR (Baisre, 2017). The proximity of CAN to JR and the higher degree of human impact present an opportunity to examine the differences in biogeochemistry and microbiology between these two Cuban reef‐systems.

The reef‐system of the Florida Keys (FK) is located in close proximity to JR and CAN, but has experienced more anthropogenic impacts relative to the Cuban reef‐systems. Reefs within FK (spanning ~570 km) are situated close to developed land within FK and South Florida and development activities have influenced the water quality in these waters (Lapointe and Clark, 1992; Lapointe *et al*., 2004). Additionally, FK hosts 2–3M tourists annually (Leeworthy *et al*., 2010), and many of these visitors engage in water activities such as boating, fishing, and scuba diving. The health of these reefs has been declining precipitously since they were first studied: algal phase shifts, eutrophication of the water column with decreases in water quality, pollution, high prevalence and spread of coral diseases, and loss of coral cover have afflicted these reefs (Szmant and Forrester, 1996; Lapointe *et al*., 2004; Precht *et al*., 2016). Additionally, commercial and recreational fishing have overexploited over 50 species of predatory fish within FK (Ault *et al*., 1998; Ault *et al*., 2006). To combat these stressors, FK was designated as a national marine sanctuary in 1990 and separated into distinct marine zones. Fishing and harvesting of any marine life are prohibited in only a small portion of these zones and public access to the reefs for recreational fishing and diving is allowed in most areas.

We designed this study to identify field‐based microbial signatures of the protected and relatively healthy reef‐system of JR and to describe how biogeochemistry and reef seawater microbial communities change along a gradient of human impact. We expected to observe microbialized reefs within FK compared to the reef‐systems in Cuba. Additionally, we hypothesized that there would be small‐scale differences in biogeochemistry and microbial community composition within each reef‐system because we included reefs along potential hydrogeographic gradients and different distances from land in order to obtain an understanding of this variability.

## Results

**Figure 1:**
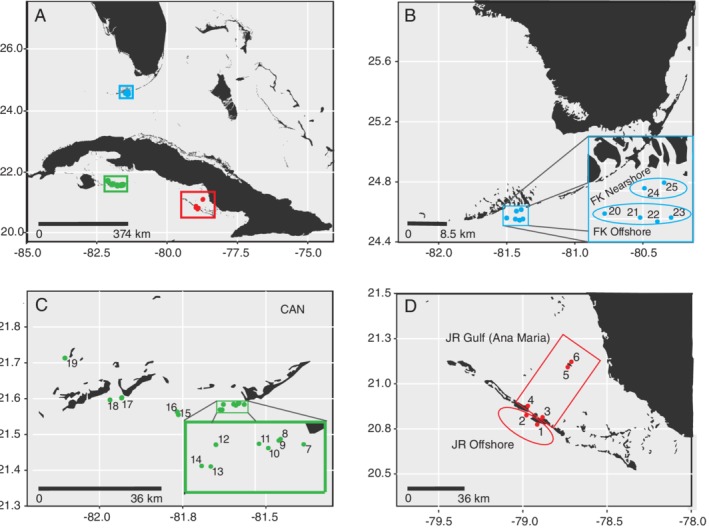
Overview map of the reef‐systems (A) and reef locations studied: (B) Florida Keys (FK), Florida, USA, (C) Los Canarreos (CAN), Cuba, and (D) Jardines de la Reina (JR), Cuba. Outlined shapes in (B) and (D) delineate the two subregion reef groupings within the Florida Keys (nearshore and offshore) and Jardines de la Reina (offshore and gulf).

Water sampling and reef surveys were conducted at reef locations across the three reef‐systems: Jardines de la Reina, Cuba (JR; 6 reefs), Los Canarreos, Cuba (CAN; 13 reefs—reef surveys were conducted at 5 reefs) and Florida Keys, USA (FK; 6 reefs) (Fig. 1, Table S1). The sampled reefs were grouped into five different subregions—JR offshore, JR gulf (Ana Maria Gulf), CAN, FK offshore and FK nearshore—*a priori* to capture spatial, environmental and anthropogenic (when applicable) gradients across each reef‐system. At each reef, divers surveyed the reef composition and then sampled surface (<1 m) and reef‐depth seawater (within 1 m of the reef).

### *Reef composition*

**Figure 2:**
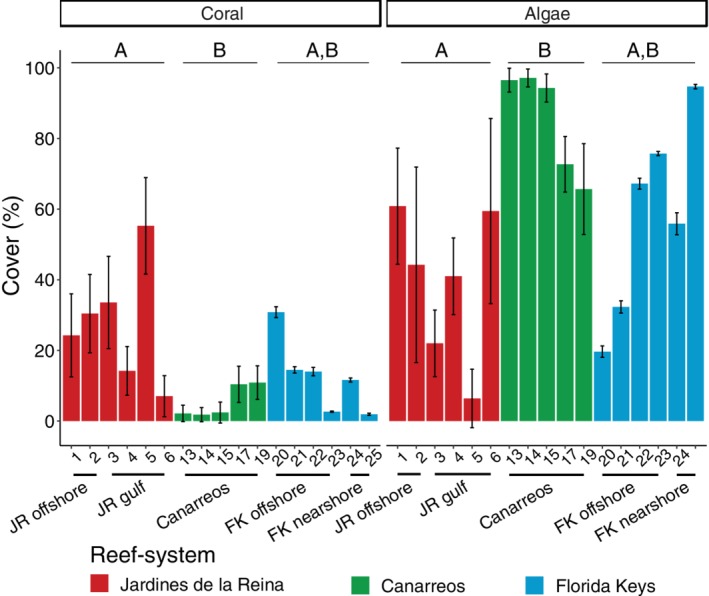
Comparison of mean coral and algal cover of the reefs indicates that Jardines de la Reina (JR) and the Florida Keys (FK) have similar coral and algal cover, whereas the Los Canarreos (CAN) reef‐system has significantly lower coral cover and higher algal cover. Reef‐systems with different letters are significantly different from each other (Kruskal–Wallis rank‐sum test, Dunn's test with Bonferroni corrections; ANOVA, Tukey's multiple comparisons of means test; *p* < 0.05) and are indicated by the letters A and B. Error bars reflect standard deviation in percent cover.

By reef‐system, average living coral cover was significantly higher [analysis of variance (ANOVA), *F*(2,14) = 4.89, *p* = 0.025] in JR and FK compared to CAN and was similar between JR and FK (Tukey's multiple comparisons of means, adjusted *p*‐value <0.05; Fig. 2). In contrast, average total algal cover was significantly higher (ANOVA, *F*(2,14) = 5.82, *p* = 0.014) in CAN compared to JR and similar to the algal cover in FK (Tukey's multiple comparisons of means, adjusted *p*‐value <0.05; Fig. 2). Reef composition also varied locally within each reef‐system (Fig. 2). Within JR, the offshore forereefs (1 and 2) had average coral and algal covers of 27.4% and 52.5% respectively (Fig. 2, Table S2). Site 5 within the JR gulf had 55.3% coral cover, the highest measured in this study. In CAN, the average coral cover among the five surveyed reefs was 5.4% and the algal cover was 85.3% (Fig. 2, Table S3). Of the six reefs surveyed in FK, the nearshore site (25) had the highest macroalgal cover (26.0%) as well as the highest total cover of algae (94.7%) out of all the Florida sites and the lowest live coral cover (1.9%) out of all of the reefs surveyed (Fig. 2, Table S2).

### *Macronutrients*

**Figure 3:**
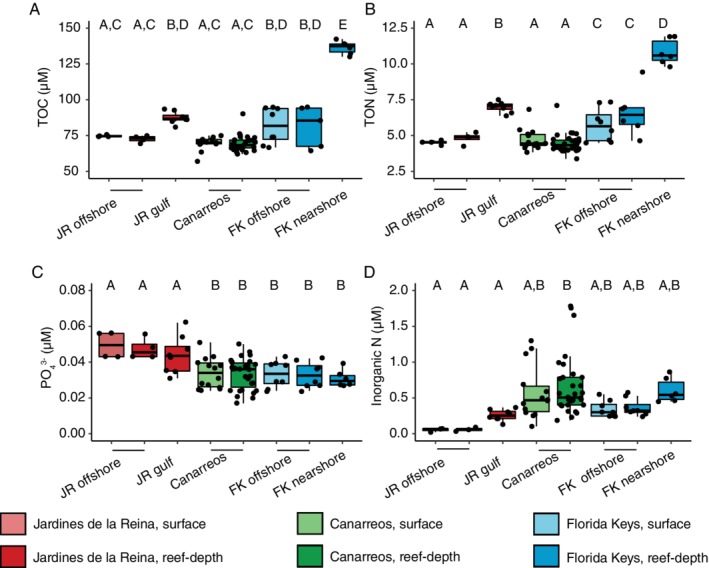
A comparison of seawater organic matter and nutrient concentrations across the reefs shows differential concentrations between the reef‐systems. Measurements include (A) total organic carbon (TOC), (B) total organic nitrogen (TON), (C), phosphate (PO_4_^3−^) and (D) inorganic nitrogen (NO_2_^−^ + NO_3_^−^ + NH_4_^+^). Lower and upper edges of the boxplot correspond to the first and third quartiles, the whiskers extend to the largest or smallest value at 1.5 times the interquartile, and the black bar across the box represents the median. Boxplots with different letters denote concentrations that are significantly different from each other (ANOVA, Tukey's HSD test, *p* < 0.05) within each plot.

The concentrations of organic and inorganic macronutrients were measured at all sites in seawater collected from surface and reef‐depths. The sampling location of reef‐depth seawater varied across sites ranging from an average depth of 5.1 m (0.75–16 m range) in JR, 5.2 m (1–14 m) in CAN, and 3.8 m (1–6 m) in FK (Table S1, Supporting Information Methods). Concentrations of total organic carbon (TOC) (ANOVA, *F*(7,27) = 78.19, *p* < 0.05), total organic nitrogen (TON) (ANOVA, *F*(7,74) = 53.44, *p* < 0.05), and dissolved inorganic nitrogen (nitrite, nitrate and ammonium) (ANOVA, *F*(7,74) = 4.21, *p* < 0.05) were significantly higher within the JR gulf as well as nearshore FK compared to the offshore JR reefs (Tukey's multiple comparisons of means, adjusted *p*‐value <0.05; Fig. 3, Fig. S1). Nitrate, nitrite, and ammonium were barely detectable in offshore JR reef seawater (Fig. 3, Fig. S1). Concentrations of nitrite (ANOVA, *F*(7,75) = 7.38, *p* < 0.05) and nitrate (ANOVA, *F*(7,76) = 3.39, *p* < 0.05) were significantly higher in CAN and FK compared to JR (Tukey's multiple comparisons of means, *p* < 0.05; Fig. S1). Concentrations of ammonium were similarly low across most of the reef locations and depths (Fig. S1). Concentrations of TOC, TON, and silicate (ANOVA, *F*(7,76) = 14.11, *p* < 0.05) were significantly higher (Tukey's multiple comparisons of means, *p* < 0.05) in FK nearshore seawater compared to seawater from other reefs (Fig. 3, Fig. S1). Lastly, nutrient concentrations between surface and reef‐depth seawater within each subregion were not significantly different (ANOVA, *p* > 0.05; Fig. 3, Fig. S1).

### *Microbial abundances and carbon contributions*

**Figure 4:**
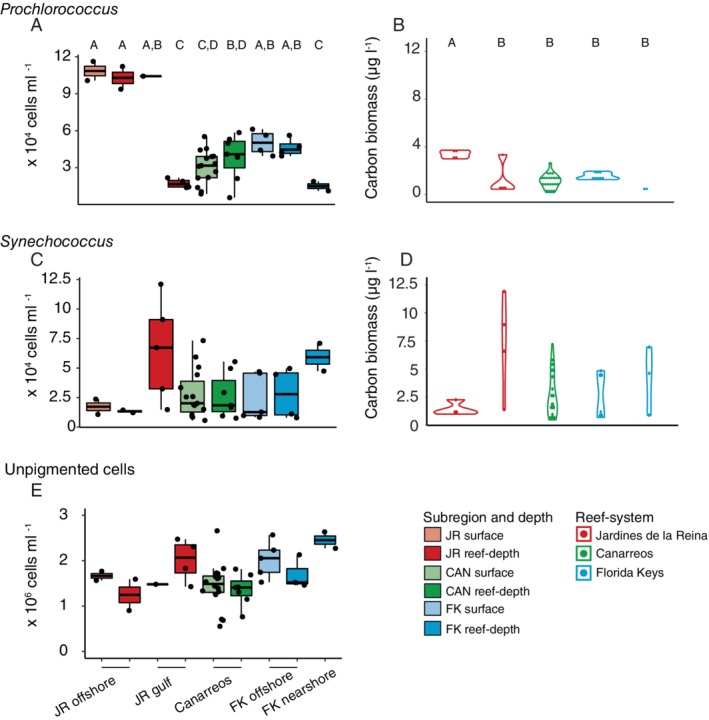
Differential microbial abundances and the carbon contributions of these cells in seawater across different reef subregions for (A, B) *Prochlorococcus* and (C, D) *Synechococcus*. Unpigmented cell abundance (largely heterotrophic bacteria and archaea) are shown in (E). Lower and upper edges of the boxplot correspond to the first and third quartiles, the whiskers extend to the largest or smallest value at 1.5 times the interquartile, and the black bar across the box represents the median. Boxplots and violin plots with different letters are significantly different from each other (Kruskal–Wallis Rank Sum test and Conover–Iman or Dunn's tests, *p* < 0.05).

Across reef‐systems, *Prochlorococcus* abundances were significantly higher (Kruskal–Wallis, *χ* ^2^ = 18.33, *df* = 4, *p* = 0.001) within JR offshore reef‐depth and surface seawater compared to JR gulf reef‐depth seawater, CAN surface and reef‐depth seawater, and nearshore FK seawater, but not significantly different from abundances in FK offshore reef‐depth and surface seawater (Conover's post hoc test, adjusted *p*‐value <0.05, Fig. 4A). In fact, *Prochlorococcus* was significantly more abundant (approximately six times higher) in offshore JR (surface and reef‐depth) as well as JR gulf surface seawater compared to JR gulf reef‐depth seawater (Fig. 4A). *Synechococcus* abundances followed the opposite pattern and were on average sixfold higher at sites located within the JR gulf compared to the offshore JR reefs but not significantly different because of variability between reefs (Fig. 4C). The abundance of unpigmented cells, generally heterotrophic bacteria and archaea, was mostly similar across all reefs and reef‐systems but elevated within reef‐depth gulf seawater (JR5 and 6) and highest within nearshore FK seawater (Fig. 4E). Picoeukaryotic cell abundances were more similar between reef‐systems (Fig. S2A). Calculations of carbon biomass demonstrated that *Synechococcus* contributed carbon biomass to all regions, with up to 12.5 μg of carbon L^−1^ in JR gulf seawater (Fig. 4D). *Prochlorococcus* contributed up to 3 μg of carbon L^−1^ in offshore JR reef seawater (Fig. 4B).

### *Phytoplankton*

**Figure 5:**
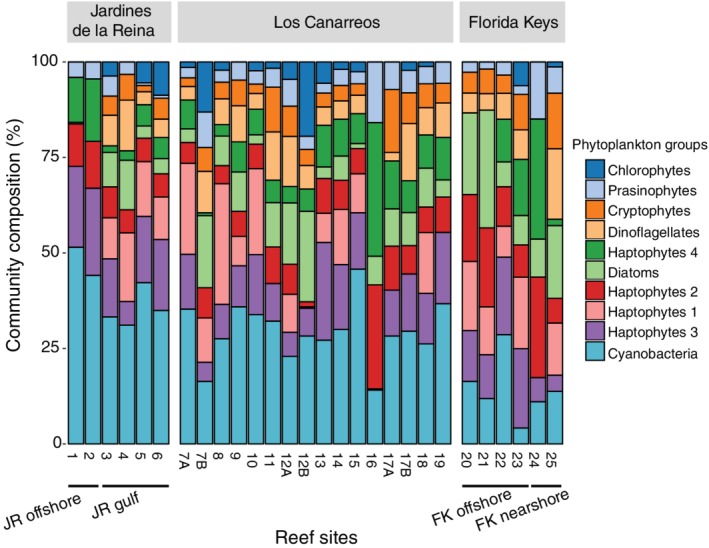
Relative abundances of phytoplankton groups across the reefs as determined by pigment analysis. Site numbers with either an A or B were sampled twice but on different days.

Chlorophyll *a* concentrations were generally low (ranging from 0.053 to 0.337 μg L^−1^) (Fig. S3) but changes in phytoplankton community composition were observed across reef‐systems. Phytoplankton community assemblages from Cuban reefs were dominated by cyanobacteria, comprising average relative abundances of 39% (JR) and 29% (CAN) of the phytoplankton community (Fig. 5). In contrast, FK reef seawater had a greater representation of the 11 other measured phytoplankton functional classes with significantly less cyanobacteria (14 ± 8%; ANOVA, *F*(2,25) = 15.98, *p* = 3.38E‐5; Tukey multiple comparisons of means, *p* < 0.05) (Fig. 5). The relative abundance of diatoms was significantly higher (ANOVA, *F*(2,25) = 4.032, *p* = 0.030) in FK (16%) compared to JR (5%) (Tukey multiple comparisons of means, *p* = 0.026) (Fig. 5).

### *Microbial alpha diversity*

**Figure 6:**
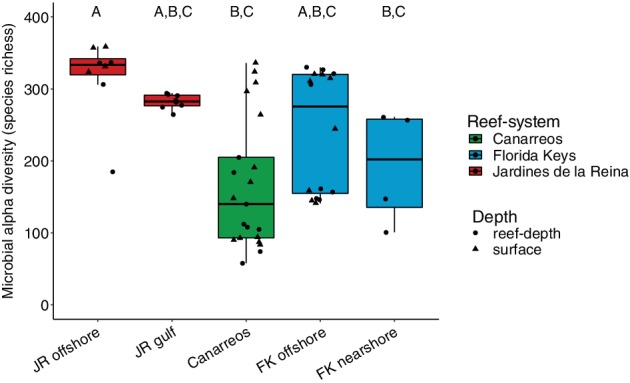
Microbial species richness (alpha diversity), as indicated by the number of bacterial and archaeal SSU rRNA gene amplicons grouped into minimum entropy decomposition (MED) nodes, is significantly highest in Jardines de la Reina offshore reef seawater compared to seawater from the other reefs. Lower and upper edges of the boxplot correspond to the first and third quartiles, the whiskers extend to the largest or smallest value at 1.5 times the interquartile, and the black bar across the box represents the median. Boxplots with different letters are significantly different from each other (Kruskal–Wallis rank‐sum test, Dunn's test using Bonferroni corrections *p* < 0.05).

Offshore reef seawater in JR had the highest microbial alpha diversity (measured here as microbial richness), as indicated by the number of minimum entropy decomposed (MED) nodes of bacterial and archaeal SSU rRNA gene amplicons [median of 333.5 (range: 185–359) MED nodes] (Fig. 6). Offshore JR seawater had significantly (Kruskal–Wallis, *χ* ^2^ = 21.41, *df* = 4, *p* = 2.6E‐4) higher alpha diversity compared to CAN (Dunn's test, adjusted *p* = 0.0001; Fig. 6) as well as FK nearshore reef seawater (Dunn's test, adjusted *p* = 0.042; Fig. 6). Offshore FK seawater had the next highest median alpha diversity [275.5 (range: 141–330) MED nodes], followed by FK nearshore reef seawater [median alpha diversity 202 (range: 101–261) MED nodes]. Microbial alpha diversity in CAN reef seawater had the lowest median richness of 140 (range: 58–336) MED nodes (Fig. 6). The variation in microbial alpha diversity between reefs within CAN as well as FK was larger compared to JR, with the largest range encountered in CAN (58–360 MED nodes) (Fig. 6).

### *Microbial community composition*

**Figure 7:**
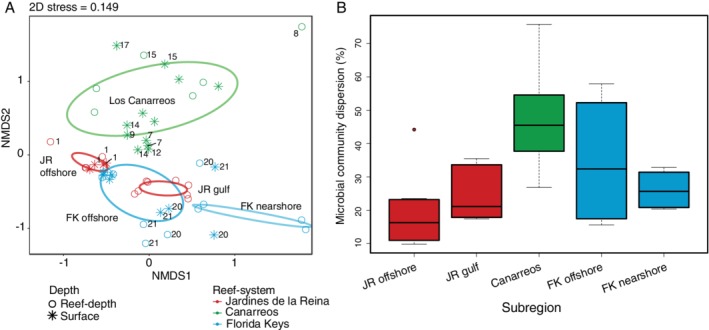
Bacterial and archaeal community beta diversity and Bray–Curtis microbial community dispersion between the reefs. A. NMDS analysis of reef seawater bacterial and archaeal SSU rRNA gene amplicons grouped into MED nodes and compared using Bray–Curtis dissimilarity. Confidence ellipses are drawn using the subregion category (using the covariance matrix), the region is indicated by colour, and depth is indicated by the symbol shape. Samples that are located outside of the covariance ellipse are labelled with the reef number where they were collected. B. Boxplots of the microbial community dispersion within each reef grouping. Lower and upper edges of the boxplot correspond to the first and third quartiles, the whiskers extend to the largest or smallest value at 1.5 times the interquartile, and the black bar across the box represents the median.

A nested permutational multivariate analysis of variance (PERMANOVA; Adonis) test on the Bray–Curtis dissimilarity index of reef seawater bacterial and archaeal SSU rRNA gene amplicons grouped into MED nodes indicated that region (reef‐system; JR, CAN, or FK), subregion, reef location, and sampling depth influenced the composition of reef seawater communities, with reef location contributing the most to variation in community dissimilarity (Table 1). Additionally, a non‐metric multidimensional scaling analysis (NMDS) corroborated these results (Fig. 7A). At a broader scale, microbial communities collected from the same reef‐system and subregion were more similar to each other (Fig. 7A and B). In the NMDS, all CAN seawater microbial communities were ordinated in the positive plane of the *y*‐axis (NMDS2) and separated from JR and FK microbial communities (Fig. 7A). Surprisingly, there was a high similarity in community composition between sites 22 and 23 in FK and JR offshore forereefs (JR 1 and 2). Microbial community dispersion was higher and more variable in seawater collected from CAN and FK offshore compared to JR offshore and JR gulf seawater, indicating higher beta diversity across these subregions (Fig. 7B).

**Table 1:** PERMANOVA (analysis of variance using distance matrices, ADONIS) comparisons based on Bray–Curtis dissimilarities of seawater bacterial and archaeal SSU rRNA amplicons grouped into MED nodes collected across reef‐systems.

Factors^a^	*df* b	Sums of squares	Mean squares	Pseudo *F* statistic	*R* ^2^ c	*p*‐value (perm.)
Region	2	2.3619	1.18093	12.5082	0.18028	0.001
Water‐type (region)	2	2.3047	1.15236	12.2057	0.17592	0.001
Reef [water‐type (region)]	18	4.7583	0.26435	2.7999	0.36320	0.001
Depth {Reef [water‐type (region)]}	9	1.3157	0.14619	1.5485	0.10043	0.039
Residuals	25	2.3603	0.09441		0.18016	
Total	56	13.1009			1.000	

a Nested comparisons are denoted by parentheses; e.g. Reef [Water‐type (Region)] indicates that the factor ‘Reef’ is nested within the factor ‘Water‐type’ that is nested within the factor ‘Region’.

b df = degrees of freedom.

c *R* ^2^ = percentage of variation explained by each factor.

perm. = 999 permutations.

Within the NMDS, microbial communities sampled within JR ordinated together by location; microbial compositions from reefs JR 1 and 2 were more similar to each other than to the other communities sampled from sites located within the JR gulf (Figs 1D and 7). Compared to the other reef‐systems, all communities from JR grouped closer together and had less variance in community composition relative to CAN and FK microbial communities (Fig. 7). The pattern of ordinating by general geographic location was not as evident in microbial communities collected from CAN and FK (Fig. 7A). Microbial community composition from site 25, one of the reefs closest to Summerland Key, was more dissimilar from the other FK microbial communities (Figs 1B and 7A). Environmental variables were fitted to the NMDS ordination using vector fitting (‘envfit’ function) and this procedure indicated that picoeukaryote abundance (*R* ^2^ = 0.11, *p* = 0.040) and nitrite (*R* ^2^ = 0.33, *p* = 0.001) and silicate (*R* ^2^ = 0.16, *p* = 0.010) concentrations were significantly correlated with the ordination of microbial communities in the NMDS.

### *Regionally specific microbial taxa*

Reef seawater microbial community composition, assessed using bacterial and archaeal SSU rRNA gene amplicons, showed some variability at the level of phylum (Fig. S4). Cyanobacteria were more abundant within CAN (29.7 ± 18.3%; mean and standard deviation) compared to JR (18.5 ± 5.8%) and FK (13.2 ± 5.4%) (Fig. S4). *Prochlorococcus* sequences were 99.2%–99.6% identical to MIT9313, a low‐light ecotype of *Prochlorococcus*, and this was the only ecotype identified within the amplicon‐based survey. Bacteroidetes was most represented in FK reef seawater, with an average relative abundance of 16.8 ± 8.3%, and less abundant in JR (12.7 ± 5.7%) and CAN (8.6 ± 6.4%) (Fig. S4). Verrucomicrobia also followed the same trend as Bacteroidetes and had the highest relative abundance in FK reef seawater (2.0 ± 1.4%) compared to JR (0.4 ± 0.3%) and CAN (0.6 ± 1.6%) (Fig. S4). Euryarchaeota were detected on nearly all reefs, with average relative abundances of 1.0 ± 0.7% in JR, 0.7 ± 0.8% in CAN and 1.3 ± 1.8% in FK (Fig. S4).

**Figure 8:**
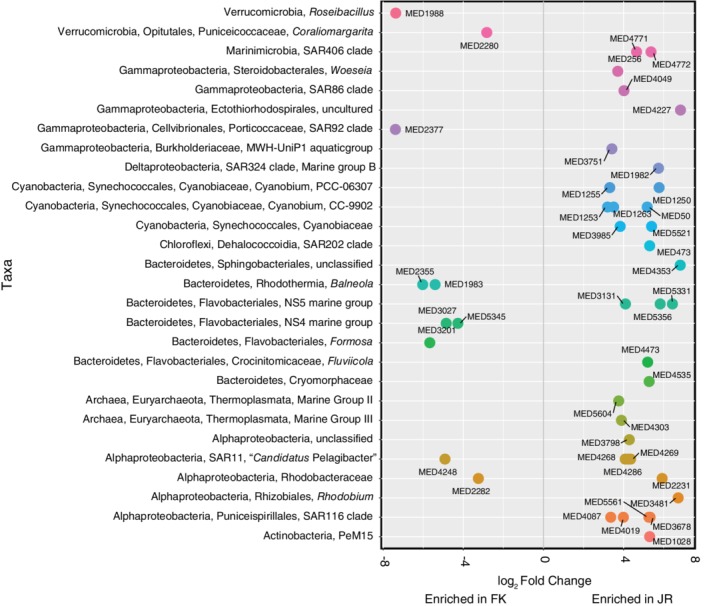
Log_2_ fold changes of the 44 significantly enriched MED nodes detected within Jardines de la Reina (JR) compared to Florida Keys (FK) reef‐depth seawater. Taxa are labelled using the highest resolved level of taxonomic annotation and colours indicate phyla or subphyla membership. Taxa identified to genus are plotted.

Enrichment comparisons of specific taxa within reef‐depth seawater collected from the most biogeochemically distinct reef‐systems of JR and FK revealed that 44 discrete MED nodes were differentially abundant (*p*‐adjusted <0.05, Benjamini–Hochberg correction for multiple testing) (Fig. 8). The 34 enriched taxa in JR belonged to microbial groups typically found in reef seawater environments. Alphaproteobacteria comprised 29% of the enriched MED nodes, including the SAR116 clade, Surface 1 and 2 groups within the SAR11 clade, and Rhodobacteraceae (Fig. 8). Cyanobacteria accounted for 20.6% of reads enriched within JR seawater, with most of the representative MED node sequences identifying as *Synechococcus*. Lastly, while the prevalence of archaea was low across the entire data set, MED nodes affiliated with Marine Groups II and III within the Thermoplasmata were significantly enriched in JR communities (5.8% of enriched sequences) (Fig. 8). MED nodes significantly depleted in JR and enriched in FK reef‐depth seawater were mostly comprised of Bacteroidetes (50%), Alphaproteobacteria (20%), and Verrucomicrobia (20%) (Fig. 8). More specifically, MED nodes affiliated with *Formosa* and *Coraliomargarita* were enriched within FK seawater compared to seawater collected from JR (Fig. 8). All of these MED nodes were present across the data set at low relative abundances (Table S3).

### *Functional differences between JR and the Florida keys*

**Figure 9:**
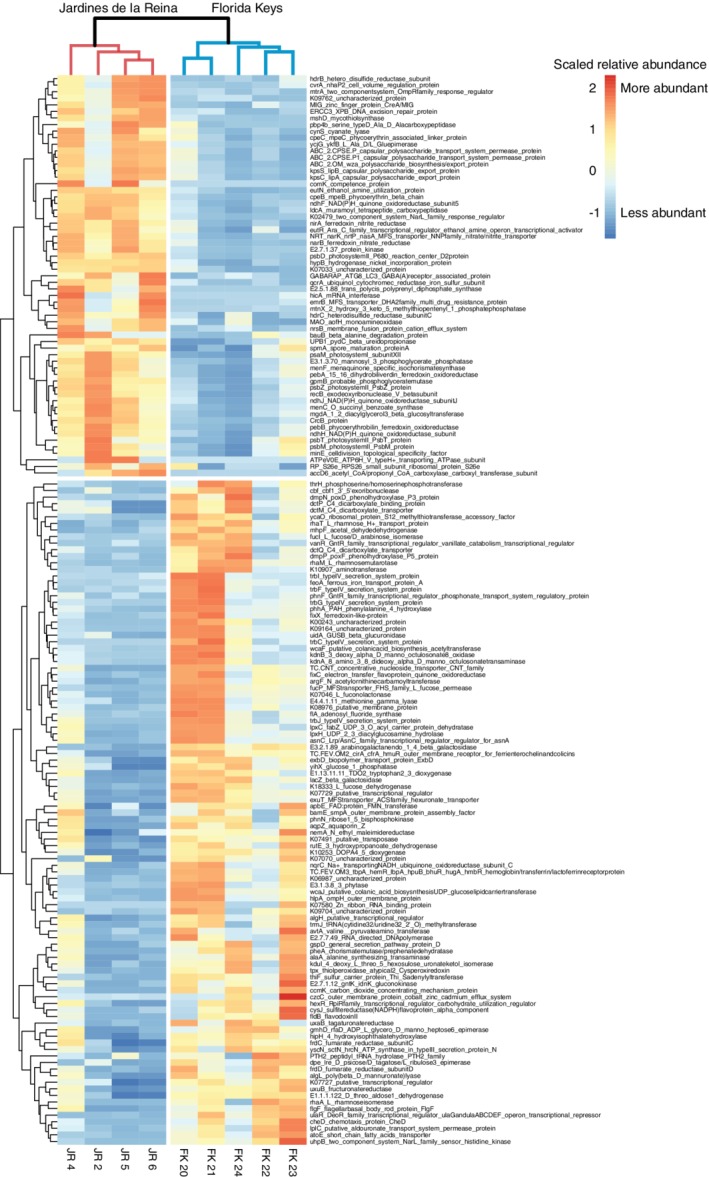
Comparison of the reef‐depth seawater metagenomes resulted in 163 significantly different KEGG orthologs (KOs) between Jardines de la Reina and the Florida Keys. KO abundances were scaled using the 10th and 90th quantiles of the data for visualization. The dendrogram reflects the hierarchical clustering of the samples using the ‘hclust’ function in R.

Metagenomic sequencing and analysis of the whole microbial community (eukaryotes, bacteria, archaea, and some DNA viruses) in reef‐depth seawater from JR and FK resulted in 163 significantly different functional genes (Fig. 9). These genes were grouped into KEGG modules as well as metabolic pathways. JR metagenomes were enriched in photosynthesis and nitrogen metabolism pathways (Fig. S7) and the KEGG modules of nitrate assimilation, assimilatory nitrate reduction, the capsular polysaccharide transport system, and the NAD(P)H: quinone oxidoreductase enzyme (for chloroplasts and cyanobacteria) (Table 2). Metabolic pathways enriched in FK included fructose and mannose metabolism, pentose and glucoronate interconversions, lipopolysaccharide biosynthesis, toluene degradation, valine, leucine and isoleucine biosynthesis, as well as the microbial metabolisms in diverse environments category (including degradation and metabolism of xenobiotics, and energy metabolism of diverse compounds) and pathway coverage ranged from 0.02 to 0.1 (Table 2). The KEGG modules that were enriched in FK included fumarate reductase and the degradation step of benzene to catechol involved in benzene degradation with module coverages ranging from 0.2 to 1 (Table 2).

**Table 2:** Enriched KEGG metabolic modules and pathways of seawater microbial communities in Jardines de la Reina, Cuba (JR, shaded in grey) and the Florida Keys, USA (FK).

	Orthology count	Reef‐system of enrichment	Coverage^a^	*p*‐value
*KEGG module definition*				
Capsular polysaccharide transport system (M00249)	2	JR	0.6667	0.0051
NAD(P)H: quinone oxidoreductase, chloroplasts and cyanobacteria (M00145)	3	JR	0.2143	0.0189
Assimilatory nitrate reduction, nitrate => ammonia (M00531)	2	JR	0.3333	0.0236
Nitrate assimilation (M00615)	1	JR	1.0000	0.0421
Fumarate reductase, prokaryotes (M00150)	2	FK	0.5000	0.0100
Benzene degradation, benzene => catechol (M00548)	2	FK	0.3333	0.0236
*KEGG pathway definition*				
Photosynthesis (map00195)	5	JR	0.0794	0.0019
Nitrogen metabolism (map00910)	4	JR	0.0667	0.0098
Oxidative phosphorylation (map00190)	7	JR (5) FK (2)	0.0326	0.0316
Pentose and glucuronate interconversions (map00040)	4	FK	0.0571	0.0166
Lipopolysaccharide biosynthesis (map00540)	3	FK	0.0750	0.0182
Toluene degradation (map00623)	3	FK	0.0652	0.0264
Valine, leucine and isoleucine biosynthesis (map00290)	2	FK	0.1053	0.0285
Fructose and mannose metabolism (map00051)	7	FK (6) JR (1)	0.0654	0.0007
Drug metabolism ‐ other enzymes (map00983)	2	FK (1) JR (1)	0.0909	0.0375
Pyruvate metabolism (map00620)	4	FK (3) JR (1)	0.0417	0.0457
Microbial metabolism in diverse environments (map01120)	22	FK (16) JR (6)	0.0204	0.0474

a Coverage indicates the normalized coverage of genes in either KEGG pathways or modules.

### *Community respiration rates*

**Figure 10:**
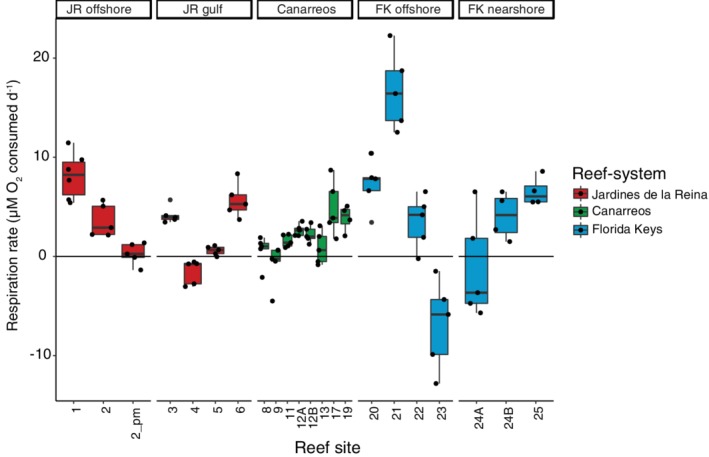
Comparison of net community respiration rates for reef‐depth seawater across reef‐systems. Lower and upper edges of the boxplot correspond to the first and third quartiles, the whiskers extend to the largest or smallest value at 1.5 times the interquartile, and the black bar across the box represents the median. Reef sites with either an A or B after the number were sampled twice but on different days. All incubations were completed with reef seawater collected in the morning with the exception of one incubation at site JR 2 that was collected in the afternoon (labelled as ‘2_pm’).

Water column net community respiration was determined by monitoring oxygen through time in dark incubations. Most of the reefs (81%) had positive community respiration rates that ranged from 0.3 to 16.7 μmol of O_2_ consumed L^−1^ day^−1^ (Fig. 10). The highest respiration rate of 16.7 μmol O_2_ L^−1^ day^−1^ was measured in offshore FK seawater collected from site 21. Negative respiration rates, implying net oxygen production, were observed in seawater collected from JR 4, CAN 9, and sites FK 23 and 24 (Fig. 10). These values ranged from 0.3 to 6.9 μmol of O_2_ produced, with the highest O_2_ production at site FK 23 (Fig. 10).

### *Across reef‐system relationships between microbial diversity, microbial abundances, and coral cover*

**Figure 11:**
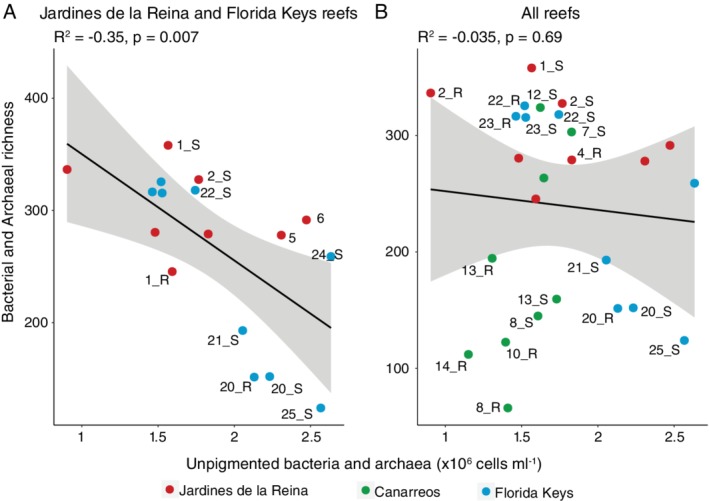
Negative regressions between bacterial and archaeal richness and unpigmented cell abundances across the three reef‐systems. Symbols that lie outside the line are labelled with the site number and depth. S denotes surface and R denotes reef‐depth samples. A. Includes only Jardines de la Reina and Florida Keys samples. B. Includes samples from all reef‐systems.

Relationships were examined across the measured parameters, and for brevity, only those with significant results are reported. There was a significant negative regression (*R* ^2^ = 0.33, *p* = 0.010, Fig. 11A) between microbial community alpha diversity and unpigmented cell abundances across JR and FK, with less microbial alpha diversity and slightly higher unpigmented abundances in FK nearshore reef seawater. However, this regression was not significant when seawater from CAN was included (*R* ^2^ = −0.02, *p* = 0.49, Fig. 11B). We also detected a significant positive regression between the abundance of picocyanobacteria (summation of *Prochlorococcus* and *Synechococcus* cell abundances) and coral cover across the reef‐systems (*R* ^2^ = 0.54, *p* = 0.001, Fig. S5).

## Discussion

This study compared reef seawater biogeochemistry and microbial communities between protected and impacted Northern Caribbean reefs with the goal of deciphering distinct microbial features. We found that JR is an oligotrophic reef‐system characterized by taxonomically diverse microbial communities with high community similarity and abundant picocyanobacterial biomass, whereas CAN and FK reefs had more spatial variability in reef seawater microbial community alpha diversity and composition. Furthermore, the spatial variability within CAN reefs may be driven by the release of nutrients from nearby wetlands (the Zapata Swamp, see below) and the hydrodynamic regime created by the complex array of cays and channels. The variability in FK may be mostly impacted by increased concentrations of organic and inorganic macronutrients, higher productivity, and/or terrestrial sources of sediments from developed land. The nearshore reefs in FK exhibited a few signs of microbialization, but this process was not as evident on the FK offshore reefs surveyed in this study. The microbial regimes observed across the reef‐systems appear to be determined by the intersection of local anthropogenic impacts as well as oceanographic processes.

### *Biogeochemical and microbial features of JR*

A majority of the macronutrient concentrations were low or barely detectable in JR and were similar to concentrations measured in other oligotrophic systems including the Sargasso Sea, the North Subtropical Pacific Gyre, the Red Sea, and other reefs in the Caribbean and Pacific, suggesting rapid turnover of these nutrient pools by microorganisms (Lewis, 1977; Westrum and Meyers, 1978; Sorokin, 1995a; Sorokin, 1995b; Karl *et al*., 1996; Dore *et al*., 2008). Organic carbon concentrations in JR (especially at JR 1 and 2) were similar to concentrations reported from a reef‐crest in Grand Cayman (Westrum and Meyers, 1978).

Nutrient dynamics across JR are likely influenced by differences in hydrodynamics between offshore forereefs and patch reefs within the JR gulf. The forereefs are flushed with pelagic, oligotrophic seawater that is carried to them by the Caribbean current, whereas the patch reefs within the gulf are influenced by productive mangrove forests and seagrass meadows that have less contact with the open ocean. Entrainment of nutrients from these productive biomes within the gulf and tidal flushing of these nutrients onto the forereefs are likely important processes that influence primary productivity, microbial diversity and metabolism, and grazing of cells by the reef community in JR.

Picocyanobacterial abundances in JR were similar to abundances observed within oligotrophic open‐ocean environments (DuRand *et al*., 2001; Zinser *et al*., 2006; Charpy *et al*., 2012) but were two orders of magnitude higher than abundances detected in seawater from Pacific reefs (Charpy *et al*., 2012). Furthermore, reef seawater collected from the offshore forereefs in JR had high abundances of *Prochlorococcus* whereas there was a shift to high, but variable abundances of *Synechococcus* in seawater collected from within the JR gulf. This negative relationship between *Prochlorococcus* and *Synechococcus* has been observed previously and tracks with increased macronutrient concentrations and proximity to land (Cox *et al*., 2006; Yeo *et al*., 2013). Additionally, the ratio of picocyanobacteria to unpigmented cells was very similar between offshore and gulf reefs in JR, potentially indicating similar nutrient or grazing controls on both populations. We expected that this change in the nutrient regime would select for different ecotypes of *Prochlorococcus*, but all the sequences identified as *Prochlorococcus* were similar to MIT9313, a low‐light adapted ecotype (Rocap *et al*., 2003). In our study, there was a discrepancy between the trends observed in *Prochlorococcus*, *Synechococcus*, and unpigmented cell abundances determined using flow cytometry and amplicon‐based relative abundances. For example, cell counts for *Prochlorococcus* and *Synechococcus* were two orders of magnitude lower than unpigmented cells, yet they still comprised a large portion of the bacterial and archaeal community based on relative abundances generated from amplicon‐based community analyses. This discrepancy likely arose because amplicon‐based sequencing data are not quantitative and cannot be directly compared with cell abundance data, as has been observed previously (reviewed within Martiny *et al*., 2009).

Despite their prevalence on reefs, the ecological roles of *Prochlorococcus* and *Synechococcus* within reef microbial communities have only been investigated in a few cases (Charpy *et al*., 2012; McDole Somera *et al*., 2016). These picocyanobacteria are some of the most important primary producers in reef seawater and they are directly and indirectly grazed by single‐celled eukaryotic heterotrophs, mixotrophic plankton, and reef organisms like corals and sponges, effectively linking photosynthetically fixed carbon from the water column to animals on the reef (Sorokin, 1995a; Sorokin, 1995b; Ferrier‐Pages and Gattuso, 1998; Bertilsson *et al*., 2005; Patten *et al*., 2011; Charpy *et al*., 2012; McNally *et al*., 2017). The high prevalence of *Prochlorococcus* and *Synechococcus* observed in this study demonstrates that picocyanobacterial dynamics on reefs should be explored further from energetic as well as community network perspectives.

Reef seawater from JR had higher microbial alpha diversity and smaller beta diversity compared to seawater from CAN and FK. There was also a negative relationship between microbial alpha diversity and heterotrophic bacterial abundance between JR and FK, indicating a potential trade‐off between community alpha diversity and biomass across the different reef‐systems. The consistent supply of oligotrophic seawater from the Caribbean current to JR forereefs likely enhances niche partitioning within microbial communities and leads to higher alpha diversity. The hydrodynamic regime likely contributes to the high microbial community similarity across this reef‐system through mixing processes. On the opposite end of the spectrum, in more disturbed and/or nutrient‐rich environments within CAN or FK, microbial alpha diversity tends to be lower or the beta diversity is higher and more variable, suggesting that disturbances on these reefs favour active growth of fewer dominant microorganisms that outcompete other cells within the population for resources (Kearns *et al*., 2016; Reese and Dunn, 2018). Additionally, genes indicative of photosynthesis and nitrogen metabolism were enriched in JR compared to FK, indicating the importance of photosynthesis and nitrogen acquisition in oligotrophic waters. Fewer genes were significantly enriched in JR compared to FK as well, suggesting a higher degree of functional redundancy and homogeneity across the more taxonomically diverse microbial communities in JR. The links between microbial alpha diversity and functional diversity continue to be debated (Louca *et al*., 2018), but our findings demonstrate that alpha diversity, in the context of reef microbial communities surveyed in JR, CAN, and FK, may be a meaningful feature of protected reefs.

### *Potential influence of nutrients from wetlands within Los Canarreos*

Reef seawater microbial beta diversity was higher and more variable in CAN compared to communities from JR and FK. In contrast, there was less variance in the inorganic and organic macronutrient concentrations, picocyanobacterial abundances, and phytoplankton community compositions across Los Canarreos. Overall, CAN reefs were less oligotrophic than the forereefs in JR and the phytoplankton community was mostly comprised of eukaryotic phytoplankton including diatoms and dinoflagellates, suggesting episodic instances of high water‐column productivity on these reefs. Additionally, the productivity of seawater microbial communities in CAN could be stimulated by nutrients and organic matter released from the Zapata Swamp, an extensive wetland (Galford *et al*., 2018) that is located ~60 km from this reef‐system.

### *Elevated nutrients near land in the Florida Keys*

Nearshore reefs in FK had the highest organic carbon, TON, and silicate concentrations compared to all the other reefs in this study. In fact, the TOC and nitrogen concentrations were 2–3 times higher on the nearshore reefs compared to the offshore reefs, on par with other observations within FK (Szmant and Forrester, 1996; Briceno and Boyer, 2015; Apprill *et al*., 2016). Terrestrial run‐off and sediment intrusion are likely partially responsible for the high TOC, TON, and silicate concentrations on these nearshore reefs, but we cannot definitively discern the relative contributions of sediment versus biological productivity because we did not measure sediment load. Despite the elevated organic carbon concentrations, community respiration rates were not higher but more variable than rates measured in reef seawater from JR and FK.

The most notable differences in reef seawater microbial community composition between JR and FK included the decrease and absence of *Prochlorococcus* cells on FK offshore and nearshore reefs, increase in the relative composition of Bacteroidetes, and detection of *Roseibaccilus* and *Coraliomargarita*, both members of the Verrumicrobia phylum, across all FK reefs. Bacteroidetes have been associated with marine particles and detected at high relative abundances following phytoplankton blooms (Pinhassi *et al*., 2004; Teeling *et al*., 2012). Furthermore, Bacteroidetes can degrade high‐molecular‐weight polymers as well as synthesize adhesion proteins for attaching to particles (Fernandez‐Gomez *et al*., 2013). Verrumicrobia are also particle‐associated, although they can be free‐living, and typically recovered from terrestrial soils (Bergmann *et al*., 2011; Freitas *et al*., 2012). That being said, Verrumicrobia are also detected ubiquitously in seawater and at high relative abundances in coastal marine environments (Freitas *et al*., 2012). Higher abundances of Bacteroidetes and Verrumicrobia suggest that there are more particles in FK seawater compared to JR and CAN, which corresponds with higher nutrient availability and shifts in phytoplankton community composition.

In FK, we observed higher total chlorophyll *a* concentrations and phytoplankton populations mostly comprised of diatoms, dinoflagellates, and haptophytes. The increased macronutrient concentrations likely enhance the growth of larger eukaryotic phytoplankton and select against the growth of microbial cells that are not tolerant of higher nutrient conditions. Additionally, there were more diverse functional metabolic pathways enriched in FK compared to JR, in agreement with the premise that microbial communities living in environments with more substrates available will have the functional capability to use the available nutrients. Furthermore, genes involved in the pentose‐phosphate pathway have been positively correlated with the algal cover on microbialized reefs (Haas *et al*., 2016) and we detected enrichment of this pathway (pentose and glucuronate interconversions) in FK. Microorganisms using the pentose‐phosphate pathway can potentially catabolize more diverse carbon sources, including carbohydrates released by algae (Haas and Wild, 2010), and this strategy has been shown to provide a selective advantage to microorganisms that need to grow faster than their competitors (Haas *et al*., 2016). We did not detect significant enrichment of virulence‐associated or pathogenic genes in seawater from FK compared to JR, which is contrary to other studies that have observed an increase in the abundance of these genes with reef degradation or increased human impact (Dinsdale *et al*., 2008; Bruce *et al*., 2012; Kelly *et al*., 2012; Kelly *et al*., 2014; Moreira *et al*., 2015).

### *Revisiting the microbialization hypothesis in the context of different reef regimes*

We hypothesized that there would be significant increases in the abundances of unpigmented cells (heterotrophic bacteria and archaea), enhanced community respiration, higher concentrations of inorganic and organic macronutrients, and shifts from coral to algal dominance on the reefs along the gradient of human impact. However, we did not observe significant changes in most of these parameters. Overall, hydrogeography and subregion were the largest influences contributing to reef similarity. Offshore reefs in both JR and FK were oligotrophic, had high abundances of picocyanobacteria, high microbial alpha diversity, and more constrained microbial beta diversity, although the magnitudes of the contrasts were different within each reef‐system. The only reefs that supported some of the predictions of the DDAM model were the nearshore reefs in FK. These two nearshore reefs in FK had significantly higher concentrations of organic macronutrients, very low abundances of *Prochlorococcus*, and significant enrichment of particle‐associated and copiotrophic microbial taxa. Our observations indicate that the process of microbialization on reefs may be more nuanced and that there are additional aspects of hydrogeography that impact these processes, resulting in different reef regimes. In this study, we surveyed a spectrum of reef regimes across JR, CAN, and FK, but we recognize that some subregions (e.g. JR offshore, JR gulf, FK nearshore) have fewer data points compared to the other categories due to sampling limitations and that care needs to be taken when interpreting the statistical differences between subregions. That being said, measurements from these locations were similar within each subregion and are likely representative of the environmental conditions. However, future studies would benefit from collecting samples at a higher spatial or temporal resolution in order to unravel the process of microbialization on reefs. Additionally, there are other examples of reefs that are subjected to high loads of organic and inorganic nutrients as well as pollution, like Vardero reef in Colombia (Pizarro *et al*., 2017) or reefs subjected to upwelling events (Leichter *et al*., 2003; Stuhldreier *et al*., 2015). Comparisons of microbial community dynamics between these drastically different reef regimes like Vardero reef with JR would extend our knowledge of how microbial communities contribute to energy cycling and reef health.

### *Relating and applying back to the reef*

Coral and algal coverage varied locally (also observed by Caballero Aragón *et al*., 2019), but did not change drastically across reef‐systems, indicating that these metrics may not be the most immediate and sensitive measure of reef health. Additionally, our observations of coral and algal cover are in agreement with another study examining coral diversity and cover on reef‐systems surrounding the island of Cuba (González‐Díaz *et al*., 2018). Furthermore, coral cover on JR reefs was lower than the historical baseline of ~50% cover in the Caribbean (Gardner *et al*., 2003) and there were observations of bleaching and coral disease, indicating that even the remote reefs of JR are impacted by environmental change and disease (Ferrer *et al*., 2016; González‐Díaz *et al*., 2018). In addition to coral cover, other aspects of reef composition, including taxonomic or functional compositions of corals, macroalgae and turf algae, and macro‐invertebrates, can serve as important metrics of reef health (Smith *et al*., 2016). Fish abundances and diversity are also used as metrics for reef health and other studies have found that abundances of commercially valuable and larger fish are higher on some reefs located within the JR National Park (Pina‐Amargos *et al*., 2014; Valdivia *et al*., 2017). That being said, we are lacking an understanding of how the diversity and abundance of fish correlate with reef biogeochemistry and microbial ecology and this should be addressed by future studies.

Reef microbial ecology may instead be a more immediate and sensitive measure of reef health than coral cover or vertebrate abundance. A growing body of research has introduced the concept of using microorganisms as bioindicators on reefs (reviewed within Glasl *et al*., 2017) as well as to predict changes in environmental conditions (Glasl *et al*., 2019) and the research presented here builds upon this knowledge. We have demonstrated that the microbial signatures of high alpha diversity, high community similarity, and high prevalence of *Prochlorococcus* may be important indicators for reef managers and restoration specialists to acknowledge. For example, there is a significant interest in restoring reefs by outplanting coral colonies onto existing reefs. While general oceanographic conditions are sometimes considered when defining sites for these efforts, reef seawater microbial ecology is not typically factored into these site decisions. As additional data sets like the one presented here emerge and we further link microbial dynamics to reef health, microbial ecology may become a more prevalent and defining consideration for reef restoration efforts.

## Methods

### *Reef surveys and sample collection*

We conducted two separate research expeditions to JR (February 2015) and CAN/FK (April/May 2015) during the Caribbean dry season. Due to sampling limitations within JR, we were only able to survey and collect samples from two reef sites in the JR offshore subregion and four reefs within the JR gulf subregion. Scuba divers conducted reef surveys at all JR and five CAN reef locations to assess the percent cover of different reef organisms and substrates (Supporting Information Methods). Coverage of a wider diversity of biotypes was assessed on FK reefs using the same methods but with a different research team (Supporting Information Methods).

At each reef location, hydrographic profiles of the water column were obtained and seawater from surface (<1 m depth) and reef‐depth (~ 1 m above reef) was collected for a variety of different analyses (Table S1, Supporting Information Methods). Seawater (4 L from each depth) was collected using a submersible groundwater pump (Mini‐monsoon sampling pump, Proactive Environmental Products) and replicate 2 L samples were each filtered onto 0.22 μm pore size, 25 mm Supor® filters (Pall Corporation). Filters were stored in cryovials, flash‐frozen in liquid nitrogen, transported in a dry‐shipper in the field, and stored at −80 °C until DNA was extracted. Additionally, 20 L of seawater from each site was filtered onto 0.1 μm pore size, 142 mm Supor filters for shotgun metagenomic sequencing. Smaller‐volume (1 ml) seawater samples were collected and preserved with 1% PFA (final concentration) for flow cytometry (Supporting Information Methods). Seawater (2–4 L) was also filtered onto 25 mm Whatman® GF/F glass microfiber filters (GE Healthcare Life Sciences) for phytoplankton pigment analyses. Seawater samples were collected in duplicate from both depths at each reef for analyses of organic and inorganic macronutrients (Supporting Information Methods).

### *Macronutrient analysis*

Total non‐purgeable organic carbon (TOC) and total nitrogen (TN; organic and inorganic) concentrations were measured using a Shimadzu TOC‐V_CSH_ TOC analyser (Hansell and Carlson, 2001). Concentrations of inorganic macronutrients (PO_4_^3−^, NO_2_^−^ + NO_3_^−^, NO_2_^−^, NH_4_^+^, silicate) were analysed using a continuous segmented flow system (as described within Apprill and Rappé, 2011). Nitrate (NO_3_^−^) concentrations for each sample were calculated by subtracting the NO_2_^−^ concentration from the NO_2_^−^ + NO_3_^−^ concentration. TON concentrations were calculated by subtracting the sum of the inorganic nitrogen species concentrations (NO_2_^−^ + NO_3_^−^ and NH_4_^+^) from the TN concentrations for each sample.

### *Phytoplankton pigments*

Pigment analysis was conducted using high‐performance liquid chromatography (Van Heukelem and Thomas, 2001). The Chemtax addition to the R‐package limSolve (Soetaert *et al*., 2009), based on the program CHEMTAX (Mackey *et al*., 1996), was used to estimate the algal composition of chlorophytes, cyanobacteria, diatoms, cryptophytes, dinoflagellates, haptophytes 1‐4, and prasinophytes within each sample based upon the concentrations of 12 different pigments (Pinckney *et al*. 2015). The initial pigment ratio matrix used to evaluate phytoplankton composition was taken from Pickney *et al*. (2015). The converged initial pigment ratio matrix was used because the phytoplankton assemblages in these samples were not determined with a microscope.

### *Cell abundances*

Samples collected for cell counts were analysed with an Altra flow cytometer (Beckman Coulter) and a laser excitation wavelength of 488 nm. Unstained and stained (SybrGreen I, Invitrogen™) subsamples were analysed to estimate the abundance of picocyanobacteria (*Prochlorococcus*, *Synechococcus*), picoeukaryotes, and unpigmented cells (proxy for heterotrophic bacteria and archaea) (Marie *et al*., 1997), respectively. Fluorescence spectra were binned, analysed, and transformed into abundances using FlowJo (v. 6.4.7) software. Total abundances of cells per sample were calculated by adding the abundances for each picoplankton class together. Estimates of the carbon biomass were calculated by multiplying the abundances of *Prochlorococcus*, *Synechococcus*, and unpigmented cells by a carbon conversion factor and then converting the concentration of carbon to micrograms per litre of seawater. The carbon conversion factors for each cell type included: 30 fg of carbon per *Prochlorococcus* cell, 200 fg of carbon per *Synechococcus* cell, and 10 fg of carbon per unpigmented cell (Fukuda *et al*., 1998; Cermak *et al*., 2017).

### *Microbial community sequencing and analysis using 16S rRNA gene surveys*

DNA was extracted from filters using two different DNA extraction methods in order to increase DNA yield (Santoro *et al*., 2010; Urakawa *et al*., 2010). DNA was extracted from duplicate samples taken at each site and depth to assess reproducibility between samples. Purified DNA from the two different extraction methods was pooled per sample using the Genomic DNA Clean and Concentrator kit (Zymo Research Corporation), quantified using the Qubit 2.0 HS dsDNA assay (ThermoFisher Scientific), and screened for quality using gel electrophoresis (1% TBE agarose gel) and the HyperLadder™ 1 kb marker (Bioline) as a size reference. DNA extraction and pooling controls (9) were also created to control for potential contamination from reagents. Lastly, genomic DNA from a microbial mock community (BEI Resources, NIAID, NIH as part of the Human Microbiome Project: Genomic DNA from Microbial Mock Community A (Even, Low Concentration), v3.1, HM‐278D) was included in the final sample array to account for amplification and sequencing error.

Extracted DNA was amplified and sequenced at the W. M. Keck Center for Comparative and Functional Genomics (University of Illinois, Urbana, IL). Briefly, V4 hypervariable regions of the 16S rRNA gene were amplified using the Fluidigm® microfluidics quantitative PCR platform and prepared for 2 × 250 bp paired‐end Illumina MiSeq sequencing (Supporting Information Methods). The Fluidgim V4 primer set 515F‐Y: 5′‐GTGYCAGCMGCCGCGGTAA (Parada *et al*., 2016) and 806RB: 5′‐GGACTACNVGGGTWTCTAAT (Apprill *et al*., 2015), accompanied with Illumina adapters, index, pad, and linker sequences, were used for amplification (Kozich *et al*., 2013). Primer‐sorted and demultiplexed sequences were quality‐filtered using mothur v.1.36.1 (Schloss *et al*., 2009). Forward and reverse reads were united and locus‐specific forward and reverse primers were removed. Reads with ambiguous positions or exceeding 275 bp in length were removed. Next, unknown, mitochondria, or eukaryotic sequences were identified (method = ‘knn’) using the Silva database v119 (Quast *et al*., 2013) and removed. UCHIME (Edgar *et al*., 2011) was used to identify and remove chimeric reads (reference = self). Sequences detected in the DNA extraction and pooling controls are believed to originate from amplicon contamination during sample processing or cross‐talk between multiplexed samples during sequencing (Wright and Vetsigian, 2016) due to their classification as marine bacteria (unclassified Rhodobacteraceae, Flavobacteria, and SAR11). To be conservative, these sequences were removed from all samples (146,540 reads; 3% of data set, accounting for 107 MED nodes). This removal occurred prior to subsampling so that it had a minimal impact on subsequent analyses. Mock community samples were removed from the data set prior to read clustering and analysed separately. The sequencing error rate was 0.0027. Sequences were then subsampled to 8500 reads per sample to minimize the impacts of uneven sequence coverage across samples but retain as many samples within the data set as possible. Sequences were clustered into biologically meaningful groups (MED nodes) using Minimum Entropy Decomposition (Eren *et al*., 2015). Sequences representing each MED node were classified in mothur (Silva v119, method = ‘knn’) and this information, along with the read counts and relative abundances, was used for microbial community analyses (Supporting Information Methods). Raw sequences were deposited in the NCBI Sequence Read Archive (SRA) under BioProject PRJNA517146.

### *Metagenomic sequencing*

Total genomic DNA was extracted from nine reef‐depth seawater samples from JR (*n* = 4) and FK (*n* = 5) using a modified cetyl‐trimethylammonium bromide—phenol:chloroform:isoamyl alcohol extraction (Supporting Information Methods). No samples from CAN were chosen because the DNA extracted from CAN yielded 16S rDNA sequences that were highly variable between sites. Genomic libraries were prepared using the Hyper Library construction kit (Kapa Biosystems, Wilmington, MA, USA) and sequenced at the W. M. Keck Center using 2 × 150 bp paired‐end Illumina HiSeq 4000 sequencing. Fastq files were demultiplexed, and library adaptors were trimmed from the 3′ ends of the reads (Supporting Information Methods). BBTools (Bushnell, 2016) was used to remove residual sequence adaptors (ktrim = r k = 23 mink = 11 hdist = 1 tpe tbo) as well as trim reads using the Phred algorithm (qtrim = rl trimq = 10). The program FMAP (Kim *et al*., 2016) was used to assign KEGG orthologs to the metagenomic reads using DIAMOND (Buchfink *et al*., 2015) as well as identify significantly different KEGG orthologs, KEGG pathways, and KEGG operons between JR and FK reef‐depth seawater metagenomes (Kruskal–Wallis test; *p*‐value <0.05, FDR adjusted to control for false positives). Raw files can also be found in the NCBI SRA under bioProject PRJNA517146.

### *Community respiration measurements*

Seawater samples (4–6 per site) were collected from reef‐depth using a submersible groundwater pump and kept in the dark. Respiration rate incubations (~24 h) were conducted with ~5× replication with seawater collected from 19 reefs in acid‐cleaned 60 ml glass Biological Oxygen Demand bottles with glass stoppers. Incubations using seawater from sites 2, 12, and 24 were conducted twice. Acid‐washed bottles were equipped with oxygen optode ‘dot’ sensors (PreSens) affixed to the glass using food‐grade silicone adhesive. In the laboratory, the perimeter of the glass stopper was filled with water using a squeeze bottle to prevent gas exchange between the water in the bottle and the atmosphere. The concentration of oxygen in the bottles was measured over time using a handheld Fibox 4 (PreSens). Incubations were conducted in a static water bath in a darkened cooler located inside a darkened room at as close to *in situ* temperatures as possible in the remotefield work locations. The incubation temperatures were 26.6 ± 0.5 °C (JR), 25.0 ± 0.2 °C (CAN), and 26.5 ± 0.5 °C (FK). Initial oxygen measurements were taken every hour (h) for the first 4 h and then approximately every 4 h after that. Ten oxygen measurement readings were taken for each incubation bottle at each timepoint. Prior to calculating respiration rates, oxygen data were quality controlled to remove any individual readings greater than one standard deviation from the mean value at a given timepoint. Linear fitting to the time course oxygen data was done in matlab (v. v7.13, MathWorks) using the ‘polyfit’ function.

### *Statistical analyses*

Due to the scope and breadth of this complex and nuanced data set with sampling limitations, we implemented different statistical tests suitable for each data set (e.g. inorganic nutrients, cell abundances, microbial community analyses) and tested for significance across different qualitative (e.g. subregion, reef‐system) and quantitative (e.g. TOC concentrations) parameters. To compare differences in reef cover, macronutrient concentrations, and cell abundances, data were inspected for normality using Shapiro–Wilk normality tests. Normally distributed data were tested using ANOVA followed by post hoc Tukey multiple comparisons of means tests using a 95% family‐wise confidence level (adjusted *p* value <0.05). For data that were not normally distributed, Kruskal–Wallis rank‐sum tests, followed by either Dunn's or Conover–Iman tests using Bonferroni corrections were used to assess significant differences (adjusted *p*‐value <0.05). We recognize that JR offshore and JR gulf subregions have fewer data points compared to the other categories, but measurements from these locations were similar within each subregion and are likely representative of the environmental conditions. We performed linear regressions using ggplot2, geom_smooth, and the method = ‘lm’ (Wickham, 2016) to investigate relationships between coral cover and picoplankton abundances, algal cover and TOC concentrations, and unpigmented cell abundances and bacterial and archaeal observed richness. A principal components analysis was conducted with biogeochemical, physicochemical, and microbial abundance data to assess collinearity between variables and to investigate which variables contributed to the most variation in both dimensions (Fig. S6).

Amplicon‐based microbial community statistical analyses were completed using R studio (R Core Development Team, 2017). Reads identifying as chloroplasts (average 275 ± 198 reads per sample; 3% of all subsampled sequences) were removed from the data set prior to beginning the analyses. NMDS was conducted using the Bray–Curtis dissimilarity index and covariance matrices for each group were plotted as 95% confidence ellipses using ‘vegan’ (as in Eren *et al*., 2015; Oksanen *et al*., 2017). The ‘vegan’ package was also used to calculate the multivariate homogeneity of group dispersions by subregion (function ‘betadisper’) and is defined as the average distance of group members to the group centroid (Oksanen *et al*., 2017). In addition, environmental vectors correlating maximally with each environmental variable were fit onto the NMDS ordination using the ‘envfit’ function in ‘vegan’ (*R* ^2^ value indicates the scaled correlation coefficient). Reef‐depth seawater collected from site FK 23 was omitted from the analysis because reef‐depth TOC was not collected from this site. Nested PERMANOVA (Adonis) tests, also available within ‘vegan’, were conducted using the Bray–Curtis dissimilarity index (999 permutations) (*p* < 0.05). To conduct this test, the factors of reef‐depth, reef location, and subregion were nested within the region (reef‐system). The package ‘phyloseq’ (McMurdie and Holmes, 2013) was used to calculate alpha richness. DESeq2 was used to identify significantly differently enriched MED nodes between JR and FK reef‐depth seawater using default parameters with a ‘local’ fit trend line (Love *et al*., 2014). This procedure is able to identify significantly differentially enriched taxa even if they are at low relative abundances and is useful for investigating MED‐specific differences in cryptic members of the community. Samples collected in CAN were not subjected to DESeq2 analysis due to the lower number of samples.

## Supporting information


**Appendix** S1: Supporting Information
