# Supplementary material for: Microbial signatures of protected and impacted Northern Caribbean reefs: changes from Cuba to the Florida Keys
Source: Environ Microbiol. 2019 Dec 11;22(1):499–519. doi: 10.1111/1462-2920.14870 (PMC6972988; doi:10.1111/1462-2920.14870)
Supplement: Supplementary file 1 — Appendix S1: Supporting Information [file EMI-22-499-s001.docx]

Microbial signatures of protected and impacted Northern Caribbean reefs: changes from Cuba to the Florida Keys

Laura Weber, Patricia González-Díaz, Maickel Armenteros, Víctor M. Ferrer, Fernando Bretos, Erich Bartels, Alyson E. Santoro, Amy Apprill^*^

^*^Corresponding author: [aapprill@whoi.edu](mailto:aapprill@whoi.edu)

Supporting Information

1. Supporting Methods
2. Supporting Tables (S1 - 2)
3. Supporting Figures (S1 – S7)

**Supporting Methods**

*Reef surveys*

Scuba divers conducted reef surveys at reefs within Jardines de la Reina, Los Canarreos, and the Florida Keys (Supporting Information Table S1). At all Jardines de la Reina and five Los Canarreos reefs, divers estimated the percent cover of dominant reef biotypes (macroalgae, coral, sponge, and sand) by recording the distance (cm) that each biotype directly intersected with the transect tape at each meter over a total distance of 10 m. This distance was then recorded as a percent cover of each biotype at each meter. This was done for 12-20 transects at each site. Coverage of a wider diversity of biotypes including bare rock (covered in sand, turf algae, or crustose coralline algae), clionid sponge, dead coral, fire coral, gorgonian, green zoanthid, live coral, macroalgae, palythoa, rubble/sand, sand, and sponge, was assessed at all FK reefs using the same methods, but by a different research team. In order to compare reef survey data collected in the FK with surveys completed in Cuba, the bare rock (covered with sand, turf algae, or CCA) category was added to the percent cover of macroalgae on each reef to represent the total algal cover. This decision was made because turf algae or CCA usually covers most surfaces on the reef that are not covered with reef organisms and this estimation was used to complete the surveys in Cuba.

*Hydrography and sample collection*

At each reef location, a YSI EXO Sonde (YSI Inc./Xylem Inc.) was lowered next to the boat and used to collect temperature, salinity, dissolved oxygen, and pH profiles of the water column (Supporting Information Table S1). A custom Matlab (Mathworks®) script was used to extract values from surface (1.5 m) and reef depths (Supporting Information Table S1).

To evaluate planktonic microbial biomass, 1 ml seawater samples from each site and reef depth were collected, transported back to the field laboratory on ice, preserved using 1% PFA (final concentration) for 30 minutes at 4°C, and flash frozen with liquid nitrogen. Unfiltered seawater samples (40 ml) were collected for the measurement of total non-purgeable organic carbon (TOC) and total nitrogen (TN) and these samples were acidified with concentrated phosphoric acid (70 μl) to remove inorganic carbon. Smaller volume seawater samples (30 ml) were collected and filtered using 0.22 μm, Sterivex ^™^ filter units for analysis of phosphate (PO_4_ ^3-^), nitrite and nitrate (NO_2_^-^ + NO_3_^-^), silicate (SiO_4_^4-^), nitrite (NO_2_^-^), and ammonium (NH_4_^+^) concentrations. Macronutrient samples were transported back to the field laboratory in a cooler on ice and then frozen at -20° C for long- term storage until they could be analyzed.

*Fluidigm amplification*

DNA extracts were amplified using Fluidigm microfluidic amplification. Before amplification, 2 ng of each DNA extract was combined with 4 µl of PCR mastermix (Roche High Fidelity Fast Start Kit) in a PCR plate. PCR primers were added to a second plate (50 µM each) and diluted with the Fluidigm loading reagent and water. The primers and extracts suspended within the mastermix were loaded into a primed Fluidigm 48.48 Access Array Integrated Fluidic Circuit (IFC) and the IFC was placed within an AX controller. The Fluidigm Biomark HD PCR machine was then used to amplify the DNA extracts (with no imaging). The following amplification steps and cycle numbers were used: 50 °C for 2 minutes (1 cycle); 70 °C for 20 minutes (1 cycle); 95 °C for 10 minutes (1 cycle); 95 °C for 15 seconds, 55 °C for 30 seconds, and 72 °C for 1 minute (10 cycles); 95 °C for 15 seconds, 80 °C for 30 seconds, 60 °C for 30 seconds, and 72 °C for 1 minute (2 cycles); 95 °C for 15 seconds, 55 °C for 30 seconds, and 72 °C for 1 minute (8 cycles), 95 °C for 15 seconds, 80 °C for 30 seconds, 60 °C for 30 seconds, and 72 °C for 1 minute (2 cycles); 95 °C for 15 seconds, 55 °C for 30 seconds, and 72 °C for 1 minute (8 cycles); and 95 °C for 15 seconds, 80 °C for 30 seconds, 60 °C for 30 seconds, and 72 °C for 1 minute (5 cycles). The total number of cycles in the first amplification process was 38.

After the first amplification, PCR products from each sample were collected and then diluted (1:100) in water. Diluted product (1 µl) from each sample was amplified using Illumina linkers and barcodes in 20 µl volume reactions. The PCR reaction conditions included 95 °C for 10 minutes (1 cycle); 95 °C for 15 seconds, 60 °C for 30 seconds, and 72 °C for 1 minute (15 cycles); and an extension step at 72 °C for 3 minutes. The total number of cycles for the second amplification process was 16.

PCR products were harvested from the second amplification and quantified. Amplicon regions and expected sizes were confirmed using a Fragment Analyzer (Advanced Analytics, Ames, IA). After size confirmation, PCR products were pooled into equal ratios. PCR product pools were run on a gel for size selection and the product was gel purified (Qiagen gel extraction kit). A Bioanalyzer (Agilent) was used to inspect the size and profiles of the pooled and purified PCR products.

*Metagenomic sequencing*

Four samples were chosen from Jardines de la Reina (sites 2, 4, 5, and 6), and five samples were chosen from the Florida Keys (20, 21, 22, 23, 24). Additionally, a DNA extraction control sample was sequenced to account for potential reagent contamination, but was not analyzed.

A modified cetyl-trimethylammonium bromide (CTAB) - phenol: chloroform: isoamyl alcohol extraction was used to extract DNA from half of each 142 mm filter. Cells on the filters were exposed to a series of physical, enzymatic, and chemical disruptions to enhance cellular lysis by using 3 freeze-thaw cycles, incubating the filters with proteinase-k (20 mg/ml) and lysozyme (20 mg/mL), and vortexing the filters. CTAB, an effective surfactant used for purifying DNA in the presence of polysaccharides (Clarke 2009), was added to the sample, followed by a phenol: chloroform (24:1), phenol: chloroform: isoamyl alcohol (25:24:1), phenol: chloroform (24:1) rinsing series. The aqueous phase was precipitated using molecular grade isopropanol overnight at -20 **°**C and the DNA pellet was rinsed with 70% ethanol twice before it was eluted into 50 μl of TE buffer (10 mM Tris-Cl, pH 7.5; 1 mM Ethylenediaminetetraacetic acid).

After sequencing, 274,418,737 paired reads were generated with an average read number of 27,441,874 (+/- 9,096,570) paired reads per sample. DNA fragment sizes for the seawater samples ranged from 280-700 bp while the DNA control sample had fragments ranging between 80-600 bp.

Supporting Table S1: Summary of reef descriptions and surface (S) and reef-depth (R) water column properties.

| **Region*** | **Site** # | **Depth (m)** | **Subregion** | **Reef type** | **Latitude and Longitude** | **Temperature**  **(°C)** | **Salinity**  **(psu)** | **DO+**  **(mg/l)** | **pH** |
| --- | --- | --- | --- | --- | --- | --- | --- | --- | --- |
| JR | 1 | 10 | JR offshore | back reef | 20.77453 N, -78.91517 W | S: 26.86 | 37.3 | 6.68 | 8.16 |
|  |  |  |  |  |  | R: 26.82 | 37.3 | 7.03 | 8.19 |
| JR | 2 | 17 | JR offshore | Fore-reef | 20.82598 N, -78.97931 W | S: 26.76 | 37.4 | 6.45 | 8.14 |
|  |  |  |  |  |  | R: 26.74 | 37.4 | 6.40 | 8.14 |
| JR | 3 | 2 | JR gulf | lagoon | 20.81478 N, -78.88320 W | S: 25.75 | 38.9 | 6.78 | 8.13 |
|  |  |  |  |  |  | R: 25.73 | 39.0 | 7.19 | 8.14 |
| JR | 4 | 1.5 | JR gulf | back reef | 20.87765 N, -78.97028 W | S: 24.68 | 38.9 | 6.34 | 8.11 |
|  |  |  |  |  |  | R: 24.68 | 38.9 | 6.34 | 8.11 |
| JR | 5 | 1.3 | JR gulf | back reef | 21.09232 N, -78.73354 W | S:24.91 | 39.7 | 6.62 | 8.17 |
|  |  |  |  |  |  | R: 24.91 | 39.7 | 6.62 | 8.17 |
| JR | 6 | 0.75 | JR gulf | back reef | 21.10845 N, -78.72080 W | S: 24.12 | 39.9 | 7.02 | 8.19 |
|  |  |  |  |  |  | R: 24.12 | 39.9 | 7.02 | 8.19 |
| CAN | 7 | 7 |  | deep fore-reef with wall drop-off | 21.58422 N, -81.56530 W | S: 29.45 | 37.43 | 6.29 | 8.16 |
|  |  |  | CAN |  |  | R: 29.35 | 37.4 | 6.31 | 8.16 |
| CAN | 8 | 5 | CAN | reef crest | 21.58693 N, -81.58308 W | S: 29.73 | 37.5 | 5.32 | 8.10 |
|  |  |  |  |  |  | R: 29.41 | 37.48 | 4.61 | 8.07 |
| CAN | 9 | 5 | CAN | reef crest | 21.58802 N, -81.58180 W | S: 28.68 | 37.37 | 5.56 | 8.06 |
|  |  |  |  |  |  | R: 28.69 | 37.38 | 5.50 | 8.07 |
| CAN | 10 | 15 | CAN | deep fore-reef | 21.58158 N, -81.59057 W | S: 27.94 | 37.36 | 6.41 | 8.12 |
|  |  |  |  |  |  | R: 27.93 | 37.36 | 6.39 | 8.15 |
| CAN | 11 | 4 | CAN | reef crest | 21.58462 N, -81.59720 W | S: 28.82 | 37.40 | 5.54 | 8.14 |
|  |  |  |  |  |  | R: 28.83 | 37.41 | 5.45 | 8.14 |
| CAN | 12 | 3 | CAN | reef crest | 21.58408 N, -81.62805 W | S: 28.82 | 37.41 | 5.75 | 8.14 |
|  |  |  |  |  |  | R: 28.82 | 37.41 | 5.72 | 8.14 |
| CAN | 13 | ~7 | CAN | deep fore-reef | 21.56855 N, -81.63165 W | S: 28.18 | 37.31 | 6.04 | 8.12 |
|  |  |  |  |  |  | R: 27.63 | 37.40 | 5.98 | 8.14 |
| CAN | 14 | 9 | CAN | deep fore-reef | 21.56893 N, -81.63820 W | S: 28.18 | 37.37 | 6.08 | 8.11 |
|  |  |  |  |  |  | R: 27.85 | 37.37 | 6.14 | 8.15 |
| CAN | 15 | 10 | CAN | Fore-reef, 500m off reef crest | 21.55521 N, -81.76323 W | S: 28.07 | 37.34 | 6.36 | 8.14 |
|  |  |  |  |  |  | R: 28.08 | 37.35 | 6.47 | 8.19 |
| CAN | 16 | ~1 | CAN | back reef | 21.56272 N, -81.76676 W | S: 28.03 | 37.39 | 6.22 | 8.12 |
|  |  |  |  |  |  | R: 27.91 | 37.36 | 6.16 | 8.15 |
| CAN | 17 | ~1 | CAN | back reef | 21.60300 N, -81.93300 W | S: 27.23 | 37.40 | 5.9 | 8.10 |
|  |  |  |  |  |  | R: 27.20 | 37.41 | 5.96 | 8.12 |
| CAN | 18 | ~1 | CAN | back reef | 21.59684 N, -81.96867 W | S: 26.81 | 37.38 | 5.43 | 5.40 |
|  |  |  |  |  |  | R: 26.82 | 37.37 | 5.40 | 8.09 |
| CAN | 19 | 10 | CAN | mid-depth fore-reef | 21.71333 N, -82.10417 W | S: 27.62 | 37.39 | 6.10 | 8.12 |
|  |  |  |  |  |  | R: 27.63 | 37.41 | 6.03 | 8.13 |
| FK | 20 | 6 | FK offshore | mid-channel patch reef | 24.55945 N, -81.50098 W | S: 27.59 | 37.36 | 6.33 | 8.20 |
|  |  |  |  |  |  | R: 27.59 | 37.36 | 6.34 | 8.21 |
| FK | 21 | 7 | FK offshore | offshore patch reef | 24.55228 N, -81.43700 W | S: 27.12 | 37.32 | 6.58 | 8.19 |
|  |  |  |  |  |  | R: 27.12 | 37.32 | 6.55 | 8.21 |
| FK | 22 | 6 | FK offshore | Spur and groove reef | 24.54500 N, -81.40600 W | S: 27.35 | 37.26 | 6.14 | 8.16 |
|  |  |  |  |  |  | R: 27.35 | 37.26 | 6.11 | 8.17 |
| FK | 23 | 6 | FK offshore | reef flat | 24.55228 N, -81.38130 W | S: 27.26 | 37.22 | 6.28 | 8.19 |
|  |  |  |  |  |  | R: 27.26 | 37.22 | 6.28 | 8.19 |
| FK | 24 | 1 | FK nearshore | nearshore reef | 24.60548 N, -81.42930 W | S: 27.98 | 37.25 | 6.65 | 8.23 |
|  |  |  |  |  |  | R: 27.98 | 37.25 | 6.64 | 8.23 |
| FK | 25 | 1 | FK nearshore | nearshore patch reef | 24.61565 N, -81.39390 W | S: 28.42 | 37.42 | 5.11 | 8.13 |
|  |  |  |  |  |  | R: 28.42 | 37.43 | 5.04 | 8.14 |

*JR = Jardines de la Reina, Cuba; CAN = Los Canarreos, Cuba; FK = Florida Keys, USA.

+ DO = dissolved oxygen

Table S2. Average percent cover of dominant reef organisms and substrates at reef sites across Jardines de la Reina, Los Canarreos, and the Florida Keys.

| **Biotype Category**  **Jardines de la Reina** | **Average % Cover (S.D.)**  **Site 1** | **Site 2** | **Site 3** | **Site 4** | **Site 5** | **Site 6** |
| --- | --- | --- | --- | --- | --- | --- |
| coral | 24.3 (11.8) | 30.4 (11.1) | 33.6 (13.1) | 14.2 (6.9) | 55.3 (13.7) | 7.0 (5.8) |
| algae | 60.9 (16.4) | 44.2 (27.7) | 22.0 (9.4) | 41 (10.9) | 6.4 (8.3) | 59.5 (26.2) |
| sand | 2.9 (3.9) | 0.6 (1.3) | 26.3 (21.0) | 39.8 (15.9) | 24.0 (12.8) | 18.3 (21.4) |
| sponge | 9.7 (6.2) | 6.4 (4.9) | 13.7 (5.9) | 4.1 (2.3) | 8.2 (5.3) | 1.9 (1.6) |
| **Los Canarreos** | **Site 13** | **Site 14** | **Site 15** | **Site 17** | **Site 19** | |
| coral | 2.2 (2.3) | 1.8 (2.0) | 2.4 (3.0) | 10.4 (5.1) | 10.9 (4.8) | |
| algae | 96.5 (3.4) | 97.2 (2.5) | 94.3 (4.0) | 72.7 (7.9) | 65.7 (12.9) | |
| sand | 0.1 (0.02) | 0 | 0.3 (0.7) | 13.2 (8.9) | 11.8 (6.5) | |
| sponge | 0.9 (1.5) | 0.7 (1.6) | 1.4 (2.1) | 0.9 (1.4) | 1.3 (1.2) | |
| **Florida Keys** | **Site 20** | **Site 21** | **Site 22** | **Site 23** | **Site 24** | **Site 25** |
| bare rock (w/ sand, turf, or CCA) | 19.7 (1.6) | 33.7 (1.3) | 63.6 (1.4) | 74.1 (0.6) | 41.4 (0.0) | 68.7 (1.1) |
| clionid sponge | 0 | 0.73 (0.16) | 0 | 0 | 0 | 0 |
| dead coral | 13.3 (1.1) | 5.6 (0.8) | 2.1 (0.2) | 0.3 (0.1) | 6.5 (0.9) | 0 |
| fire coral | 0.1 (0.0) | 0.4 (0.1) | 0.6 (0.1) | 1.0 (0.1) | 0.1 (0.0) | 0.4 (0.1) |
| gorgonian | 3.8 (0.3) | 3.4 (0.1) | 1.8 (0.2) | 2.4 (0.1) | 1.4 (0.1) | 0.5 (0.1) |
| green zoanthid | 0 | 0 | 0.2 (0.05) | 0 | 0 | 0 |
| live coral | 30.8 (1.5) | 14.5 (0.9) | 14.0 (1.2) | 2.7 (0.2) | 11.6 (0.6) | 1.9 (0.3) |
| macroalgae | 0 | 1.4 (0.1) | 3.6 (0.3) | 1.7 (0.1) | 14.5 (0.7) | 26.0 (1.0) |
| palythoa | 0 | 1.5 (0.2) | 11.9 (0.7) | 0 | 0 | 0 |
| rubble/sand | 6.2 (0.5) | 8.7 (1.7) | 0.5 (0.1) | 2.8 (0.4) | 7.9 (1.8) | 1.2 (0.4) |
| sand | 21.3 (2.0) | 19.9 (1.3) | 0 | 2.4 (0.3) | 12.0 (2.1) | 1.1 (0.2) |
| sponge | 4.9 (0.3) | 10.2 (0.3) | 1.8 (0.1) | 5.3 (0.2) | 4.6 (0.5) | 0.2 (0.0) |
| total algae* | 19.6 (1.6) | 32.3 (1.7) | 67.2 (1.5) | 75.7 (0.6) | 55.9 (3.2) | 94.7 (0.6) |

S.D. = standard deviation

*total algae = average of the sum of macroalgae and bare rock (w/ sand, turf, or CCA) categories

Table S3. Relative abundances (%) and standard deviations of significantly enriched (grey shading) or depleted MED nodes in Jardines de la Reina or Florida Keys reef seawater as revealed by DESeq2.

| MED node | Taxonomy | Mean (SD) JR | Mean (SD) FK |
| --- | --- | --- | --- |
| MED1988 | Verrucomicrobia, *Roseibacillus* | 0 | 0.30 (0.60) |
| MED2280 | Verrucomicrobia, Opitutales, Puniceicoccaceae, *Coraliomargarita* | 0.10 (0.12) | 1.03 (0.96) |
| MED4771 | Marinimicrobia, SAR406 clade | 0.11 (0.14) | 0.01 (0.02) |
| MED4772 | Marinimicrobia, SAR406 clade | 0.090 (0.14) | 0.001 (0.006) |
| MED256 | Gammaproteobacteria, Steroidobacterales, *Woeseia* | 0.06 (0.06) | 0.0070 (0.016) |
| MED4049 | Gammaproteobacteria, SAR86 clade | 0.12 (0.14) | 0.013 (0.035) |
| MED4227 | Gammaproteobacteria, Ectothiorhodospirales, uncultured | 0.313 (0.440) | 0.0036 (0.0091) |
| MED2377 | Gammaproteobacteria, Cellvibrionales, Porticoccaceae, SAR92 clade | 0 | 0.40 (0.53) |
| MED3751 | Gammaproteobacteria, Burkholderiaceae, MWH-UniP1 aquatic group | 0.10 (0.12) | 0.030 (0.08) |
| MED1982 | Deltaproteobacteria, SAR324 clade, Marine group B | 1.09 (0.76) | 0.04 (0.05) |
| MED1255 | Cyanobacteria, Synechococcales, Cyanobiaceae, Cyanobium, PCC-06307 | 0.27 (0.24) | 0.02 (0.05) |
| MED1250 | Cyanobacteria, Synechococcales, Cyanobiaceae, Cyanobium, PCC-06307 | 0.027 (0.33) | 0.005 (0.012) |
| MED1253 | Cyanobacteria, Synechococcales, Cyanobiaceae, Cyanobium, CC-9902 | 0.26 (0.27) | 0.04 (0.05) |
| MED1263 | Cyanobacteria, Synechococcales, Cyanobiaceae, Cyanobium, CC-9902 | 0.07 (0.08) | 0.0080 (0.015) |
| MED50 | Cyanobacteria, Synechococcales, Cyanobiaceae, Cyanobium, CC-9902 | 0.074 (0.14) | 0 |
| MED3985 | Cyanobacteria, Synechococcales, Cyanobiaceae | 0.077 (0.075) | 0.01 (0.01) |
| MED5521 | Cyanobacteria, Synechococcales, Cyanobiaceae | 0.13 (0.21) | 0.0050 (0.013) |
| MED473 | Chloroflexi, Dehalococcoidia, SAR202 clade | 0.16 (0.16) | 0.0040 (0.11) |
| MED4353 | Bacteroidetes, Sphingobacteriales, unclassified | 0.18 (0.34) | 0 |
| MED2355 | Bacteroidetes, Rhodothermia, *Balneola* | 0.0090 (0.16) | 0.72 (0.95) |
| MED1983 | Bacteroidetes, Rhodothermia, *Balneola* | 0 | 0.29 (0.70) |
| MED3131 | Bacteroidetes, Flavobacteriales, NS5 marine group | 0.06 (0.08) | 0.004 (0.008) |
| MED5356 | Bacteroidetes, Flavobacteriales, NS5 marine group | 0.11 (0.16) | 0.0030 (0.012) |
| MED5331 | Bacteroidetes, Flavobacteriales, NS5 marine group | 0.19 (0.23) | 0.004 (0.0130) |
| MED3027 | Bacteroidetes, Flavobacteriales, NS4 marine group | 0.014 (0.021) | 0.53 (0.81) |
| MED5345 | Bacteroidetes, Flavobacteriales, NS4 marine group | 0.016 (0.019) | 0.24 (0.29) |
| MED3201 | Bacteroidetes, Flavobacteriales, *Formosa* | 0.0018 (0.0072) | 0.14 (0.23) |
| MED4473 | Bacteroidetes, Flavobacteriales, Crocinitomicaceae, *Fluviicola* | 0.089 (0.14) | 0.00071 (0.0032) |
| MED4535 | Bacteroidetes, Cryomorphaceae | 0.79 (0.83) | 0.018 (0.043) |
| MED5604 | Archaea, Euryarchaeota, Thermoplasmata, Marine Group II | 0.11 (0.16) | 0.0064 (0.0098) |
| MED4303 | Archaea, Euryarchaeota, Thermoplasmata, Marine Group III | 0.11 (0.12) | 0.0010 (0.020) |
| MED3798 | Alphaproteobacteria, unclassified | 0.045 (0.058) | 0.00066 (0.0029) |
| MED4248 | Alphaproteobacteria, SAR11, "*Candidatus* Pelagibacter" | 0.0018 (0.0049) | 0.070 (0.13) |
| MED4268 | Alphaproteobacteria, SAR11, "*Candidatus* Pelagibacter" | 0.038 (0.056) | 0.0014 (0.0044) |
| MED4286 | Alphaproteobacteria, SAR11, "*Candidatus* Pelagibacter" | 0.14 (0.22) | 0.0064 (0.019) |
| MED4269 | Alphaproteobacteria, SAR11, "*Candidatus* Pelagibacter" | 0.14 (0.17) | 0.013 (0.023) |
| MED2282 | Alphaproteobacteria, Rhodobacteraceae | 0.19 (0.30) | 1.90 (2.12) |
| MED2231 | Alphaproteobacteria, Rhodobacteraceae | 0.18 (0.23) | 0.0078 (0.019) |
| MED3481 | Alphaproteobacteria, Rhizobiales, *Rhodobium* | 0.94 (1.36) | 0.0099 (0.024) |
| MED4087 | Alphaproteobacteria, Puniceispirillales, SAR116 clade | 0.24 (0.19) | 0.29 (0.045) |
| MED4019 | Alphaproteobacteria, Puniceispirillales, SAR116 clade | 0.28 (0.34) | 0.0082 (0.021) |
| MED5561 | Alphaproteobacteria, Puniceispirillales, SAR116 clade | 0.12 (0.13) | 0.0014 (0.0042) |
| MED3678 | Alphaproteobacteria, Puniceispirillales, SAR116 clade | 0.073 (0.12) | 0 |
| MED1028 | Actinobacteria, PeM15 | 0.13 (0.11) | 0.0056 (0.0096) |


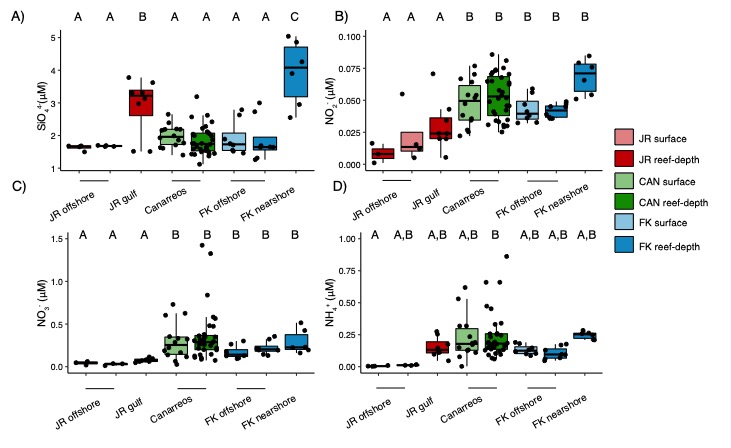


Figure S1. Concentrations of organic and inorganic macronutrients measured by subregion and reef-system. A) SiO_4_^4-^ (Silicate) B) NO_2_^-^ (nitrite), C), NO_3_^-^ (nitrate), D) NH_4_^+^ (ammonium). Boxplots are drawn as follows: the lower and upper edges of the boxplot correspond to the first and third quartiles, the whiskers extend to the largest or smallest value at 1.5 times the interquartile, and the black bar across the box represents the median. Boxplots with different letters are significantly different from each other (ANOVA, Tukey’s HSD test, p < 0.050). JR = Jardines de la Reina, CAN = Canarreos, FK = Florida Keys. Surface refers to surface seawater.

Figure S2. Cell abundances of picoplankton functional groups, including picoeukaryotes (A) and total cells (summation of *Prochlorococcus*, *Synechococcus*, Picoeukaryotes, and unpigmented cells at each depth and subregion) (B). Lower and upper edges of the boxplot correspond to the first and third quartiles, the whiskers extend to the largest or smallest value at 1.5 times the interquartile, and the black bar across the box represents the median. No significant differences were detected (Kruskal-Wallis Rank Sum test and Dunn’s test, p < 0.050).

Figure S3. Concentrations of total chlorophyll *a* by subregion and reef-system.

__

Figure S4. Relative abundance (%) of bacterial and archaeal phyla determined from SSU rRNA gene amplicons. S indicates surface and R indicates reef-depth. Replicate samples are numbered. Samples with * were collected from the same location but on a different day.


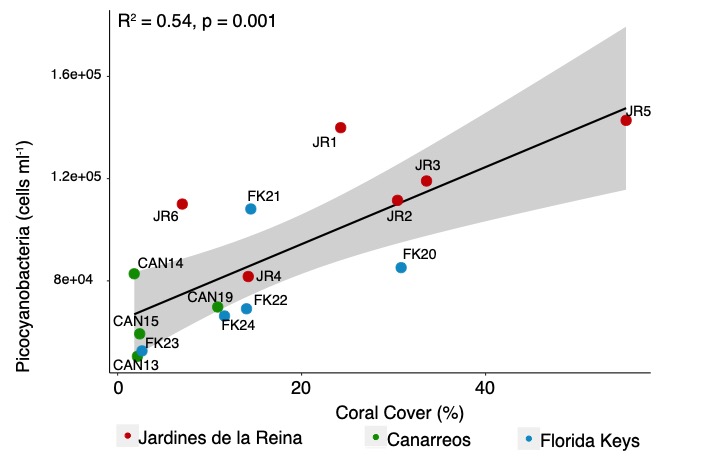
 Figure S5. Regression between the abundance of picocyanobacteria (the summation of the abundance of *Prochlorococcus* and *Synechococcus* cells) detected in reef depth seawater and coral cover across the three reef-systems. Each point is labeled with the site name.

Figure S6. Principal components analysis (PCA) biplot of the physicochemical, biogeochemical, and cell abundance measurements collected across reef-systems. Symbol color and shape reflect subregion. N.N = nitrate + nitrite, Pro = *Prochlorococcus*, Syn = *Synechococcus*, DO = dissolved oxygen, TOC = total organic carbon, TON = total organic nitrogen, TN = total nitrogen, tchla = total chlorophyll *a*, temp. = temperature.


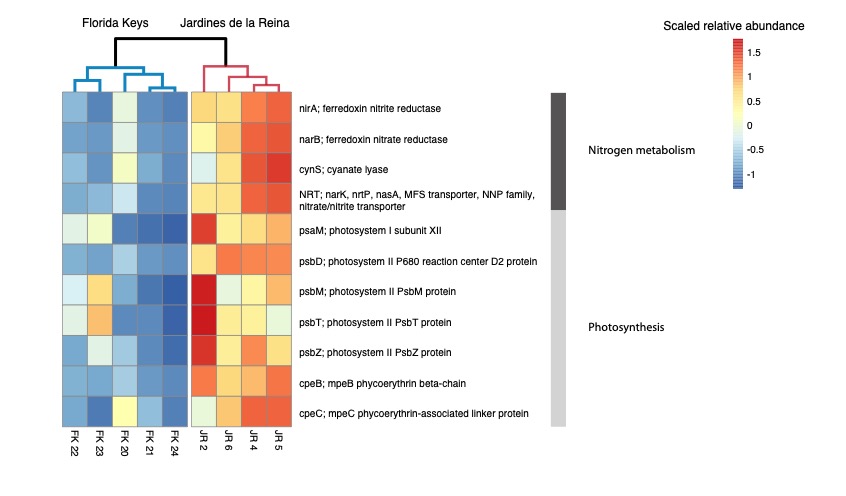


Figure S7. Photosynthesis and nitrogen metabolism genes are enriched in Jardines de la Reina compared to the Florida Keys. The abundances of KEGG orthologs (KOs) were scaled using the 10^th^ and 90^th^ quantiles of the data for visualization. The dendrogram reflects hierarchical clustering of the samples using the ‘hclust’ function in R.
